# Optimising the selection of welfare indicators in farm animals

**DOI:** 10.3389/fvets.2025.1661470

**Published:** 2025-10-28

**Authors:** Jon Day, Mohamed Ben Haddou, Rita Kylling, Guro Vasdal, Heleen van de Weerd

**Affiliations:** ^1^Chronos Sustainability Ltd., Chichester, United Kingdom; ^2^Cerebrus Advies, Dinxperlo, Netherlands; ^3^Mentis SA, Ixelles, Belgium; ^4^Matprat, Oslo, Norway; ^5^Animalia AS, Oslo, Norway

**Keywords:** animal welfare, iceberg indicators, MCDA, optimisation, welfare assessment, welfare indicator, welfare risk assessment

## Abstract

**Introduction:**

Risk assessment (RA) frameworks are increasingly being applied to improve the welfare of farmed animals. These frameworks have at their core, a logic chain linking welfare hazards (risks) with one or more welfare consequences which, in turn, are each measured by one or more welfare indicators. Effective and efficient monitoring of animal welfare often involves the selection of a subset of indicators from a large pool. Selecting ‘iceberg indicators’ could be advantageous due to their association with multiple welfare consequences. However, no standardised, data-driven method exists to select optimal combinations under practical constraints. This study addresses this gap by creating an algorithmic approach to optimise indicator selection.

**Methods:**

The work was conducted in six phases: (1) construction of a structured database of welfare indicators; (2) a proof-of-concept study; (3) design of a greedy selection algorithm; (4) enhancement of the algorithm using branch-and-bound and backtracking methods; (5) performance and sensitivity testing, and (6) creation of two case studies. A dataset of 382 animal welfare indicators across seven farm species was compiled from scientific opinions published by the European Food Safety Authority (EFSA) and from other published literature. The EFSA scientific opinions contain data acquired through a rigorous process of literature reviews and expert elicitation and consensus panels to link welfare indicators with their associated welfare hazards and welfare consequences. To enable algorithm development, the *Coverage* of each welfare indicator was first determined by calculating the number of unique welfare consequences to which it was linked. Metadata such as the *Impact of welfare consequence* [Low (1) or High (2)], *Ease of hazard mitigation* [Easy (1), Moderate (2) or Difficult (3)], and *Ease of indicator use* [Easy (1), Moderate (2) or Difficult (3)] was generated through an expert elicitation process. These data were standardised using max–min normalisation across all criteria, and an objective function was defined which enabled indicator subset selection according to various user-defined criteria. Optimisation was performed using both a greedy algorithm and an enhanced algorithm incorporating backtracking and branch-and-bound solvers. Algorithm performance and robustness were evaluated through sensitivity analyses, scenario testing, and computational benchmarking.

**Results:**

The greedy algorithm offered computational efficiency but incorporated suboptimal plateaus in *Coverage* as additional indicators were combined. The enhanced algorithm identified globally optimal combinations within 0.2 s for all species, regardless of problem size. In a broiler chicken case study, the enhanced algorithm removed indicators that were moderately difficult to use. A pig case study showed that the enhanced algorithm combined the same welfare indicators as the greedy algorithm but validated the added value of multi-criteria scoring by identifying high-impact, easy-to-implement indicators suitable for welfare certification.

**Discussion:**

The enhanced algorithm was able to move beyond the selection of iceberg indicators, by incorporating multiple selection criteria to inform welfare indicator choice. The enhanced algorithm is data-agnostic and enables users to optimise indicator selection with diverse datasets spanning research, industry, and policy contexts. Its flexibility supports the development of tailored applications for different stakeholders. Future work should explore processes to determine weighting values, scenario testing, robustness, and stakeholder engagement to maximise both relevance and practicality.

## 1 Introduction

The measurement of farm animal welfare has gained substantial prominence in research, policy and practical applications related to animal care and management. Historically, welfare has been assessed according to the housing and resources that were provided for animals (input- or resource-based measures, e.g., the size of an animal enclosure) (1). However, over time, the focus has shifted to outcome- or animal-based measures (e.g., lameness) because welfare is a characteristic of the individual animal, not just the system in which animals are kept (2). Furthermore, there has also been recognition that management inputs (animal care) profoundly influence the level of welfare achieved (3). Besides biological functioning and a focus on resources, welfare assessments now also increasingly include welfare outcomes in terms of animals’ experiences (i.e., their affective states) (4).

Farm animal welfare is not only the responsibility of the farmers who manage their day-to-day care. For example, veterinarians collaborate with farmers to safeguard animal health and welfare. Industry bodies and levy-funded organisations work to promote animal welfare in their sectors. Third-party certification schemes and animal welfare labels often necessitate additional inspections that provide confidence to food businesses (processors, retailers, restaurants and bars) and citizens (5). Investors and banks both seek to ensure that standards of animal welfare do not pose financial risks (6). Policy makers and national competent authorities create and enforce legal frameworks for the protection of animals (7). Despite their differing priorities, all these stakeholders need objective and quantifiable indicators to monitor the welfare of animals across diverse agricultural production systems.

The European Union (EU) uses scientific and technical evidence as a foundation for its legislation and policies on animal welfare (as a component of feed and food safety). The European Commission has mandated the European Food Safety Authority (EFSA) to provide this scientific support. EFSA has developed a Risk Assessment (RA) framework for animal welfare to facilitate effective, evidence-based policy making. This framework includes several steps, namely hazard identification, hazard characterisation, exposure assessment and risk characterisation for a specified housing system, and is broadly based on the methodology already established in human and animal health (8). The RA framework has at its core, a logic chain linking welfare hazards (risks) with one or more welfare consequences which, in turn, are each measured by one or more welfare indicators (9, 10).

A welfare hazard is an exposure variable causing a risk to animal welfare. A welfare consequence is a consequence of a welfare hazard and impairs the welfare of the animals. Welfare indicators are observations and measures made during a welfare assessment.

The EFSA panel on Animal Health and Welfare (AHAW) has published numerous scientific opinions which apply the RA framework (11). Each scientific opinion was written by a suitably qualified team of experts which acquired data using a rigorous and systematic approach. This approach involves reviewing published literature, conducting expert elicitation processes and discussion through consensus groups. The most recent scientific opinions include recommendations on which welfare indicators can be used to measure the welfare of the main farmed animal species.

Given the extensive range of potential animal welfare indicators, choosing the most appropriate ones to address specific assessment objectives can be challenging. This choice is further complicated by the existence of different scoring systems and weighting of welfare indicators across systems/protocols. Therefore, the selection process must carefully consider numerous factors such as the validity of the indicators in reflecting actual animal welfare, their reliability and consistency across differing contexts, the feasibility of measurement across practical scenarios, and the associated resource and cost implications (12). From a practical point of view, a comprehensive, on-farm multi-criteria welfare assessment can also take a significant amount of time (13). Therefore, it could also be beneficial to create a decision support tool that reduces the number of animal welfare indicators to be measured without losing valuable information on animal welfare.

This concept of iceberg indicators was first defined as: *“key welfare indicators that can reflect, or are closely correlated with, a range of other welfare indicators”* (14). An iceberg indicator provides an overall assessment of welfare, just as the protruding tip of an iceberg signals its submerged bulk beneath the water’s surface (ibid.). FAWC used the presence of an intact pig tail at slaughter as a simple example. The intact tail indicates the absence of both tail docking and tail biting. This can be taken to infer that the animal’s husbandry and management were managed to a sufficient level to avoid tail biting.

The definition of what constitutes an iceberg indicator in the context of the EFSA risk-based framework is stated in the latest scientific opinions from the AHAW panel. For example, the scientific opinion on broiler welfare stated: *“Some of the ABMs are relevant to more than one welfare consequence (iceberg indicators) and can be used for general welfare screening purposes, often used to get an impression of the welfare status of a flock”* (15). The scientific opinion on laying hen welfare stated: *“Animal-based measures that are relevant to more than one welfare consequence are referred to as ‘iceberg indicators’”* (16). The opinion on pig welfare stated: *“The animal-based measures that help to identify more than one welfare consequence are preferred. These indicators are commonly referred to as ‘iceberg indicators’”* (10).

These definitions highlight the important question of how closely different welfare indicators are correlated. In the development of a decision support tool, it is essential to visualise how welfare measures interact, particularly the connections between animal-based indicators and the environmental factors that influence them (12). Knowledge of such interactions could facilitate the identification of the optimum combination of measures. For example, if a large set of indicators possesses overlapping information (such as related welfare hazards or welfare consequences), it may be possible to identify a smaller set of measures that have the same explanatory power. The RA framework for animal welfare presents an ideal basis on which to analyse and visualise the interactions between welfare hazards, welfare consequences and welfare indicators.

This study used an existing database that collated welfare measurement data from multiple sources, including several EFSA scientific opinions on the welfare of farmed animals. The database included a list of welfare indicators and the associated welfare hazards and welfare consequences. In the present paper, these data were further enriched with categorical metadata on dimensions such as the *Impact of welfare consequences*, *Ease of hazard mitigation*, and *Ease of indicator use.*

The database was used to develop algorithms and compare methods of discovering optimal combinations of welfare indicators while maintaining the link with related hazards and consequences. The objective of the work was to enable users to select combinations of welfare indicators, subject to user-defined constraints/inputs, that meet specific criteria such as reflecting multiple welfare consequences, being easy to deploy, and having the potential to mitigate the most severe impacts of certain welfare hazards.

## 2 Materials and methods

The research detailed in this paper was conducted in six phases: (1) database building, (2) a proof-of-concept study, (3) development of a greedy algorithm, (4) enhancement of the greedy algorithm using backtracking and branch-and-bound methods and (5) assessment of algorithm functionality and (6) generation of case studies to illustrate how the algorithms could be used in practice. The following sections provide further details on the methodology followed in each phase.

### 2.1 Database building

In 2022, MatPrat (the Norwegian Egg and Meat Council) catalogued the indicators that are available to measure the welfare of selected food-producing animals in Norwegian systems of production (Table 1). The project aimed to gain insights to focus animal welfare activities, for example, to use in dialogue with the Norwegian industry.

**Table 1:** A description of the species and systems of production represented in the test dataset.

Species	Class (e.g., system of production / life-stage)
Pigs	Meat pigs
	Pregnant sows - Groups
	Lactating sows - Pens
	Unweaned piglets
	Boars
Broiler chickens	Indoor (with or without veranda)
Laying hens	Laying hens - Aviaries
	Laying hens - Aviaries and free range
Dairy cattle	Tie stalls
	Cubicles
Dairy calves	All
Beef cattle	Adults - Cubicles
	Adults - Tie stalls
	Beef calves
	Beef bulls
Sheep	Adults - Outside year-round
	Lambs from outside year-round
	Adults - Outside/winter housed
	Lambs from winter housed

For most species, there was more than one class (system of production or life-stage).

The main welfare hazards for each species within each housing system were collated through scientific literature reviews. In the case of the EFSA scientific opinions, these welfare hazards were often explicitly listed and could be transcribed directly. For the other sources, these data were extracted through detailed review of the published article. The resulting welfare hazards were then linked to both welfare consequences and welfare indicators. In the case of the EFSA scientific opinions, again, these linkages were often explicitly specified. For the other sources, the linkages were made by two or more welfare experts in the project team. Most data were extracted from a series of EFSA reports (published from 2007 to 2012, and the updated reports from 2022 to 2023) which described the welfare aspects for the main European categories of farmed animals and housing systems. The main data sources are summarised below:

- Dairy cattle (17–29)
- Pigs (10, 27, 29–32)
- Beef cattle (17–22, 26–29, 33–36)
- Sheep (27, 29, 37, 38)
- Broiler chickens (15, 29, 33, 39)
- Laying hens (16, 29)

The database was then supplemented with metadata generated through an elicitation process involving five farm animal welfare experts. This required rating the *Impact of welfare consequence* (Low or High), *Ease of hazard mitigation* (Easy, Moderate or Difficult), and *Ease of indicator use* (Easy, Moderate or Difficult). Where the expert ratings differed, a consensus approach was used to determine the final rating.

As an example, high stocking density was identified as a welfare hazard for growing/finishing pigs. In the database, this welfare hazard was associated with three unique welfare consequences (soft tissue lesions and integument damage; general disruption of behavior, and resting problems). These welfare consequences were associated with eight welfare indicators (body lesions; calluses and bursitis (pressure injuries); ear lesions; impaired social behavior; leg injuries; pig cleanliness; restlessness, and tail lesions). Soft tissue lesions and integument damage was rated as a welfare consequence that has a ‘High’ impact. High stocking density was rated as a hazard that is ‘Easy’ to mitigate, and tail lesions was rated as a welfare indicator that is ‘Easy’ to use.

### 2.2 Proof-of-concept study and data preparation

#### 2.2.1 Proof-of-concept study

Before proceeding to the creation of the two algorithms, a proof-of-concept study was conducted. This involved exploring the effect of different combinations of indicators on the number of linked welfare consequences. The number of unique combinations of indicators increases exponentially as more indicators are combined, therefore, the approach was validated using a subset of six indicators for broiler chickens and six for laying hens.

The six indicators for each species were selected from the top of a list that was ranked by the number of unique associated welfare consequences. This ranked list was obtained by creating a pivot table in Microsoft Excel of the number of unique (i.e., deduplicated) welfare consequences linked to each indicator. First, the maximal number of unique welfare consequences associated with a combination of all six indicators was calculated (i.e., the target). Then, to identify the simplest (smallest) subset of indicators that were associated with the maximal number of welfare consequences, the number of welfare consequences that were associated with each unique combination of indicators [*n* = (2^6^–1) = 63] was determined, again, by creating a pivot table in Microsoft Excel.

#### 2.2.2 Algorithm development

Following the proof-of-concept study, two types of algorithms were created: (a) A simple greedy algorithm, which is a method to combine indicators based purely on one or more dimensions, such as the number of associated welfare consequences (e.g., selecting the best iceberg indicators first), and (b) An enhancement of the greedy algorithm using multi-criteria decision analysis (MCDA) involving backtracking (a systematic way of exploring all combinations of indicators to find one or more valid solutions) while employing branch-and-bounds to identify intelligent ‘shortcuts’, effectively guiding the search towards the optimal solution and avoiding unnecessary computations.

Greedy algorithms were first proposed as a method to determine the shortest path or subtree to connect nodes within a network. Early algorithms were used to solve the ‘minimum connector’ or ‘travelling salesman’ problem (40, 41). Greedy algorithms make a locally optimal choice to find a globally optimal solution (56). They are some of the simplest algorithms in combinatorial optimisation and can determine efficiently the solution to many problems (57). While their key advantage is that they are easy to understand and implement, greedy algorithms ignore the possibility that the solution identified may not be the best (i.e., is not the global optimum).

As greedy algorithms may converge on a locally optimal rather than globally optimal solution, an algorithm using branch-and-bound plus backtracking was developed to iteratively enumerate all subsets of ( S ) indicators. The recursive nature of such algorithms allows for exhaustive exploration of indicator subsets while using pruning techniques to discard unfeasible solutions early.

Backtracking is a systematic method for exploring all variants of a solution to find one or more valid solutions. The use of the term backtracking was first attributed to Lehmer in the 1950s (42). Such methods incrementally explore potential solutions and backtrack if a suboptimal variant is discovered. While backtracking can identify the globally optimum solution, the required number of iterations is computationally intensive, and the required number of calculations increases exponentially with the number of branches in the decision tree. For example, the potential number of combinations of a single factor increases from 31 when combining 5 items to 1,048,575 when combining 20 items [*n* = (2^items^) – 1]. However, the backtracking method can be enhanced for combinations of a larger number of items using branch-and-bound methods.

The development of branch-and-bound methods is widely attributed to (43). While simple backtracking explores all paths in the search space until a solution is found, branch-and-bound intelligently cuts off (prunes) unproductive paths early in the search, making it much more efficient for optimisation problems. Branch-and-bound uses the power of backtracking to systematically explore solutions while employing bounds to function as intelligent ‘shortcuts’, guiding the search towards the optimal solution and avoiding unnecessary computations.

It was predicted that a greedy algorithm would provide a quick and simple means of discovering and combining iceberg indicators (based on the number of associated welfare consequences for each indicator), but this would have a risk of false solutions (i.e., local maxima). It was further predicted that refinement of the greedy algorithm using backtracking would avoid the selection of false solutions but may increase the compute time to potentially unfeasible levels, so the use of branch-and-bound methods were investigated to limit the required compute time.

#### 2.2.3 Data preprocessing and normalisation

Some data preprocessing and normalisation were necessary to facilitate the development of the algorithms. For example, the dataset contained several data structures containing categorical values (see Table 2).

**Table 2:** A description of the dimensions and levels of measurement in the test dataset for each of the seven focal species of farm animal.

Type	Dimensions	Levels (where applicable)
Hazards	Welfare hazards	
Consequences	Welfare consequences	
Metadata	Impact of welfare consequence	Low | High
Metadata	Ease of hazard mitigation	Easy | Moderate | Difficult
Indicators	Welfare indicators	
Metadata	Ease of indicator use	Easy | Moderate | Difficult

To enable the use of these data in the data analysis, selected descriptors were first mapped to numerical values. These included *Impact of welfare consequence* (Low = 1 & High = 2), *Ease of hazard mitigation* (Easy = 1, Moderate = 2, Difficult = 3), and *Ease of indicator use* (Easy = 1, Moderate = 2 & Difficult = 3).

For dimensions such as the *Impact of welfare consequence*, the objective function should increase when its values are numerically higher. In this case, the values were directly added to the objective function. However, for factors such as *Ease of indicator use* and *Ease of hazard mitigation*, the objective function should increase when their values are numerically lower. To reflect this, it was necessary to either specify those factors as being negative in the objective function, or subtract values from the number of levels transformed valued=((number of levels+1)−value)) . In the present exercise, the second option was selected (Equations 1–3), which ensured that higher values are always treated as beneficial.


(1) $${\text{ImpactScore}}_{i}=({\text{Impact of welfare consequence}}_{i})$$

(2) $${\text{MitigationScore}}_{i}=((4+1)-{\text{Ease of hazard mitigation}}_{i})$$

(3) $${\text{EasinessScore}}_{i}=((4+1)-\text{Ease of indicator}\;{\mathrm{use}}_{i})$$

As the primary objective was to optimise the number of unique welfare hazards and/or unique welfare consequences that were linked to a combination of indicators, the *Coverage* for each indicator ( i ) was calculated (Equations 4, 5).


(4) $$\begin{array}{l} {\text{HazardCoverage}}_{i}=\\ \text{number of unique hazards that indicator covers} \end{array}$$

(5) $$\begin{array}{l} {\text{ConsequenceCoverage}}_{i}=\\ \text{number of unique consequences that indicator covers} \end{array}$$

To ensure that all data could be equitably weighted, each factor was then subjected to a max-min normalisation to scale it within the range {0,…,1}. This transformation also ensured that both *HazardCoverage_i_* and *ConsequenceCoverage_i_* were expressed as a proportion of the maximum number of hazards and consequences respectively, which enabled them to be equitably weighted (Equation 6).


(6) $$\text{Normalised factor}=\frac{x_{i}-x_{\min}}{x_{\max}-x_{\min}}$$

#### 2.2.4 Definition of the objective function

Each of the algorithms had at its core an objective function that incorporated all of the factors to be optimised. To enable the separate and interactive weighting of *HazardCoverage_i_* and *ConsequenceCoverage_i_*, a composite measure for *Coverage* was calculated, which also incorporated weighting factors (*α* and *β*) to adjust the balance as required (Equation 7).


(7) $${\text{Coverage}}_{i}=(\alpha .{\text{HazardCoverage}}_{i})+(\beta .{\text{ConsequenceCoverage}}_{i})$$

Additionally, the other dimensions were incorporated into the objective function (e.g., *Ease of indicator us*e, *Impact of welfare consequence*, and *Ease of hazard mitigation*). Each factor was separately weighted to enable ‘tuning’ of the objective function in different use-cases (phase 6), (Equation 8).


(8) $$\begin{array}{l} {\text{Objective}}_{i}=\\ \omega_{\text{coverage}}(\alpha .\text{HazardCoverage}+\beta .\text{ConsequenceCoverage})\\ +\omega_{\text{easiness}}(\text{EasinessScore})+\omega_{\text{impact}}(\text{ImpactScore})\\ +\omega_{\text{mitigation}}(\text{MitigationScore}) \end{array}$$

α, β, ω_coverage_, ω_easiness_, ω_impact_ and ω_mitigation_ are user-defined weighting factors that relate to the relative importance of each constraint. For example, if a user wanted to optimise based only on the number of welfare consequences linked to a combination of indicators, they could set all other weighting factors to zero. This effectively removes those factors from the calculation.

When selecting a combination of indicators S⊆{1,…,MAXindicators} the total objective function becomes (Equation 9):


(9) $$\text{Objective}\;(S)=\sum_{i\in S}{\text{objective}}_{i}$$

#### 2.2.5 Development tools

Algorithms were developed using the Python programming language (version 3.13) and Google’s OR-Tools Linear Solver (44). OR-Tools is an open-source module which enables the deployment of linear programming (LP), mixed-integer programming (MIP), Solving Constraint Integer Programmes (SCIP), and other optimisation techniques.

### 2.3 Development of a greedy algorithm

The greedy algorithm involved the selection of a pre-determined number of welfare indicators from a list of indicators ranked by the size of the objective function (Eqn. 9). For example, if no more than six indicators should be combined, the greedy algorithm selected the indicators with the top 6 highest ${\text{objective}}_{i}$ values. The user could predefine the number of indicators to be combined according to their specific use-case (e.g., desired number of indicators ≤ 4).

### 2.4 Enhanced optimisation algorithm using SCIP

The objective of the enhanced algorithm was to identify combinations of indicators (up to a user-defined maximum number of indicators) that maximise the value of the objective function (i.e., the combination(s) with the largest sum of ${\text{objective}}_{i}$ ). To achieve this, the algorithm was created using the Solving Constraint Integer Programmes (SCIP) component of the OR-Tools Linear Solver module (45).

SCIP is one of the most powerful and versatile solvers for mathematical optimisation, especially in the case of mixed-integer programming (MIP). MIP is a type of mathematical optimisation where some decision variables have categorical values, while others may be continuous (i.e., real numbers). A MIP problem typically involves optimising a linear objective function subject to a set of linear constraints. The inclusion of integer variables allows MIP to model discrete and decision analysis for problems that involve both quantitative allocations and binary or categorical choices. The recursive nature of the algorithm, combined with branch-and-bound pruning and initialisation with a greedy solution, allows for an exhaustive but efficient exploration of indicator subsets. The approach ensures an optimal selection process within the defined constraints while significantly reducing computational overhead.

SCIP solves integer programming problems by combining several algorithms. It starts by solving the LP relaxation and, if the solution is fractional, it applies branch-and-bound: branching on fractional variables to create subproblems, solving relaxations at each node, and using bounds to prune unpromising branches. Backtracking is used to navigate the search tree when dead ends are reached. During its iterations, SCIP employs greedy heuristics to quickly find good feasible solutions, which helps improve pruning efficiency. This intelligent combination of exploration and pruning leads SCIP to the optimal integer solution efficiently.

**Figure 1:**
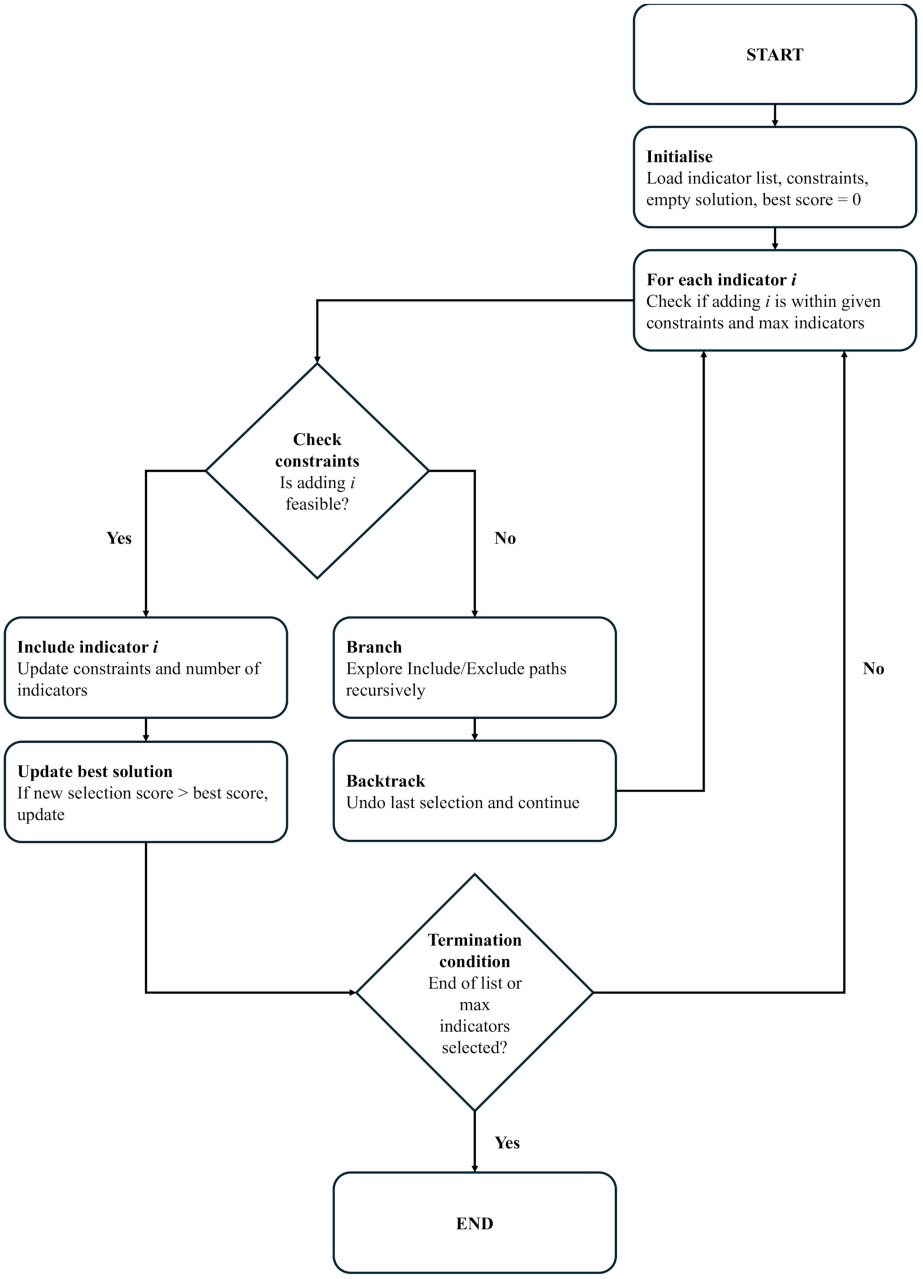
An overview of how the enhanced algorithm combined backtracking and branch-and-bound heuristics through SCIP.

In the present exercise, the algorithm operated by iterating through a list of candidate indicators, where the ‘value’ of each indicator was determined using the objective function (Eqn.9). At each step, the algorithm evaluated whether an indicator ( i ) could be included in the selection without exceeding the maximum allowable number of indicators set by the user. It explored two recursive branches: one that included the indicator (provided constraints permit) and one that excluded it. The function then backtracked by undoing previous selections to explore alternative combinations. SCIP maintained a global dictionary to store the best solution encountered during execution, updating it whenever a higher-scoring subset was identified (Figure 1).

To avoid overly lengthy computation times for larger combinations of indicators, an early stopping mechanism was incorporated by introducing a time limit parameter. If the execution time exceeded 10 s, the algorithm halted, returning the best solution identified up to that point. Additionally, progress logging was implemented at regular intervals to facilitate tracking during execution. The early stopping mechanism avoided the algorithm freezing if an overly complex set of parameters were evaluated. Additionally, as the goal was to produce an algorithm that provided solutions within practical time constraints, the early stopping mechanism helped to identify when run times had exceeded a defined threshold.

### 2.5 Assessment of algorithm functionality

Following the development of the enhanced optimisation algorithm, basic assessments of its performance and behavior were conducted under varying configurations.

First, the weighting applied to *Coverage* (ω_coverage_) was systematically varied to assess how the objective function responded to different configurations. For each level of weighting, the optimisation problem was solved, and the resulting composite objective function score was recorded. This approach enabled the decomposition of the objective function into interpretable elements, allowing direct observation of how each criterion influenced the final optimisation output.

Second, a univariate perturbation analysis was conducted to evaluate the sensitivity of the algorithm to user-defined inputs. For each weighting parameter, controlled shifts (e.g., ±50%) were introduced and the optimisation problem solved repeatedly to observe changes in the selected subset of welfare indicators.

### 2.6 Creation of case studies

To demonstrate and validate the utility of the optimisation framework, two illustrative hypothetical case studies were developed which targeted distinct user needs: (a) a food business that sought to measure six welfare indicators to demonstrate the year-on-year impact of its activities on improving the welfare of broilers in its supply chain, and (b) an animal welfare certification scheme provider that sought to measure six welfare indicators to demonstrate the welfare status of growing and finishing pigs across certified farms.

Each case study deployed both the simple greedy algorithm and the enhanced algorithm. The case studies were focused on a specific animal species and stakeholder use-case and incorporated a defined set of constraints and priorities. These included fixing the maximum number of indicators to be selected (*n* = 6), pre-defining different weighting configurations reflecting different prioritisation strategies (e.g., differential emphasis placed on *Coverage*, *Ease of indicator use*, *Impact of welfare consequence*, and *Ease of hazard mitigation*).

In each case study, the computational performance of the enhanced algorithm was investigated by recording the total runtime for increasingly complex optimisation tasks (increasing the desired number of welfare indicators in the solution). Stability of the optimisation process was further assessed by re-running the same configuration multiple times. To achieve this, the desired number of indicators was increased from 5 to 50 (in 10 steps of 5). At each step, the optimisation was run 15 times, and the mean value and 95% confidence intervals were calculated and plotted. A one-way analysis of variance was used to detect any effect of the number of indicators on computational performance, and linear regression was used to determine the direction and strength of the association between the two variables.

Scenario testing was used to explore how different weighting (prioritisation) strategies influenced the selection of welfare indicators in the enhanced algorithm. The four weighting factors were systematically varied: *Coverage*, *Ease of indicator use*, *Impact of welfare consequence*, and *Ease of hazard mitigation*. Differential weighting of these factors enabled manipulation of their relative importance in decision-making.

To ensure comparability across scenarios and maintain consistency, the sum of the weights in each scenario was fixed. Several prioritisation scenarios were designed to evaluate plausible real-world use-cases. These included *Priority on coverage*, *Balanced*, *Priority on ease of indicator use*, *Priority on impact of welfare consequence*, and *Priority on ease of hazard mitigation* (Table 3). By systematically varying the emphasis placed on each component, the scenario testing provided insight into how different stakeholders might arrive at distinct, yet justifiable, solutions depending on their operational goals and constraints.

**Table 3:** The different scenarios used to evaluate algorithm performance.

Scenario	Weight				Comment
	Coverage	Ease of indicator use	Impact of welfare consequence	Ease of hazard mitigation	
Priority on coverage	8.0	0.0	0.0	0.0	Only optimising coverage
Balanced	2.0	2.0	2.0	2.0	Equal weighting to all factors
Priority on ease of indicator use	5.0	3.0	0.0	0.0	Favouring indicators that are easy to use
Priority on impact of welfare consequence	5.0	0.0	3.0	0.0	Favouring consequences that have a large impact on welfare
Priority on ease of hazard mitigation	5.0	0.0	0.0	3.0	Favouring hazards that are easy to mitigate

The weighting for both α. HazardCoverage and β. ConsequenceCoverage, as part of overall Coverage, was held constant at 0.5, providing an equitable balance between both measures (Equation 8).

Robustness testing was used to introduce systematic perturbations in the weighting values within the objective function. This enabled investigations into the leverage that a factor exerted on the objective function. For each species of farm animal, the weighting of *Coverage*, *Ease of indicator use*, *Impact of welfare consequence* and *Ease of hazard mitigation* was manipulated by a factor of −50%, 0 or 50%, and the impact on the welfare indicators selected was recorded. All weighting factors were initially set at 1.0, except for the one that was to be manipulated. The maximum number of indicators was constrained to 10. Venn diagrams were plotted with values representing the number of shared indicators in each perturbation condition. Large variation in the welfare indicators selected by the algorithm would suggest that the weighting value (and underlying dimension) exerts a leverage on the solution obtained. Conversely, little or no variation in the welfare indicators selected by the algorithm would suggest that the weighting value (and underlying dimension) exerts no leverage on the solution obtained.

## 3 Results

### 3.1 Database

The data model was based on the RA framework developed by the European Food Safety Authority (EFSA) to describe welfare hazards, their linked welfare consequences, and the range of available welfare indicators. Data were extracted for key farmed animal species and relevant housing systems (published by EFSA between 2007 and 2012, and from the updated reports in 2022–2023). These data were supplemented with information from other scientific sources.

After data capture, the database contained 382 unique welfare indicators across a variety of farm animals dairy cows (*n* = 54); dairy calves (*n* = 51); pigs (*n* = 92); beef cattle (*n* = 48); broiler chickens (*n* = 53); laying hens (*n* = 42); and sheep (*n* = 42).

### 3.2 Proof-of -concept studies

#### 3.2.1 Broiler chickens

The top 6 welfare indicators for broiler chickens (ranked by the number of welfare consequences) were: (A) Injurious pecking, (B) Plumage damage, (C) Lethargy, (D) Footpad dermatitis, (E) Feather and body dirtiness, and (F) Walking impairment.

A combination of all six indicators was linked to a maximum of 18 welfare hazards and 9 consequences for broiler chickens. It was found that two combinations of four indicators explained the same number of welfare hazards and consequences as a combination of all six indicators (Table 4).

**Table 4:** A matrix indicating all possible unique combinations of six welfare indicators [*n* = (2^6^–1) = 63].

Number of indicators in combination					
1	2	3	4	5	6 (all)
A	AB	ABC	ABCD	ABCDE	**ABCDEF**
B	AC	ABD	ABCE	ABCDF	
C	AD	ABE	ABCF	**ABCEF**	
D	AE	ABF	ABDE	ABDEF	
E	AF	ACD	ABDF	ACDEF	
F	BC	ACE	ABEF	BCDEF	
	BD	ACF	ACDE		
	BE	ADE	ACDF		
	BF	ADF	**ACEF**		
	CD	AEF	ADEF		
	CE	BCD	BCDE		
	CF	BCE	BCDF		
	DE	BCF	**BCEF**		
	DF	BDE	BDEF		
	EF	BDF	CDEF		
		BEF			
		CDE			
		CDF			
		CEF			
		DEF			

Shaded cells indicate the combinations that were linked to the same number of welfare hazards and consequences as a combination of all six indicators for broiler chickens.

The proof-of-concept study for broiler chickens showed that the same welfare hazards and consequences can be explained more simply by measuring just four indicators: lethargy, feather and body dirtiness, walking impairment (C, E, and F), and either injurious pecking (A) or plumage damage (B) instead of using all six indicators.

The solution identified for broiler chickens highlights an important feature that may be relevant to other decision support tools. It is possible to arrive at multiple, equally valid solutions. This raises the possibility of selecting among them based on additional factors such as ease of use, time required, or cost.

#### 3.2.2 Laying hens

The top 6 indicators for laying hens (ranked by the number of welfare consequences) were: (A) Plumage damage, (B) Injurious pecking, (C) Bruises, (D) Beak shape and length, (E) Pecking wounds to the back, vent and tail, and (F) Flock records (death due to pecking wounds).

Using all six indicators explained a maximum of 14 welfare hazards and 6 consequences for laying hens. It was found that there was one combination of three indicators that explained the same number of welfare hazards and consequences as a combination of all six indicators (Table 5).

**Table 5:** A matrix indicating all possible unique combinations of six welfare indicators [*n* = (2^6^–1) = 63].

Number of indicators in combination					
1	2	3	4	5	6 (all)
A	AB	ABC	ABCD	ABCDE	**ABCDEF**
B	AC	ABD	ABCE	**ABCDF**	
C	AD	ABE	**ABCF**	**ABCEF**	
D	AE	**ABF**	ABDE	**ABDEF**	
E	AF	ACD	**ABDF**	ACDEF	
F	BC	ACE	**ABEF**	BCDEF	
	BD	ACF	ACDE		
	BE	ADE	ACDF		
	BF	ADF	ACEF		
	CD	AEF	ADEF		
	CE	BCD	BCDE		
	CF	BCE	BCDF		
	DE	BCF	BCEF		
	DF	BDE	BDEF		
	EF	BDF	CDEF		
		BEF			
		CDE			
		CDF			
		CEF			
		DEF			

Shaded cells indicate the combinations that were linked to the same number of welfare hazards and consequences as a combination of all six indicators for laying hens.

The proof-of-concept study for laying hens showed that the same welfare consequences can be explained more simply by measuring just three indicators: plumage damage (A), injurious pecking (B) and flock records (death due to pecking wounds) (F) instead of using all six indicators.

### 3.3 Greedy algorithm

It was hypothesised that animal welfare indicators vary in their *Coverage*, forming a continuum from ‘broad’ (those linked to a large number of welfare hazards and consequences) to ‘narrow’ (those only linked to a few welfare hazards and consequences). Consequently, it was expected that efforts to maximise the number of unique welfare hazards or consequences captured by combining multiple indicators would exhibit diminishing returns. For example, the first few indicators selected would contribute substantially to overall *Coverage*, while each additional indicator would contribute progressively less. As a result, when applying a greedy algorithm to optimise indicator selection, it was predicted that a saturating exponential relationship would exist between the number of indicators included and the cumulative *Coverage* achieved.

**Figure 2:**
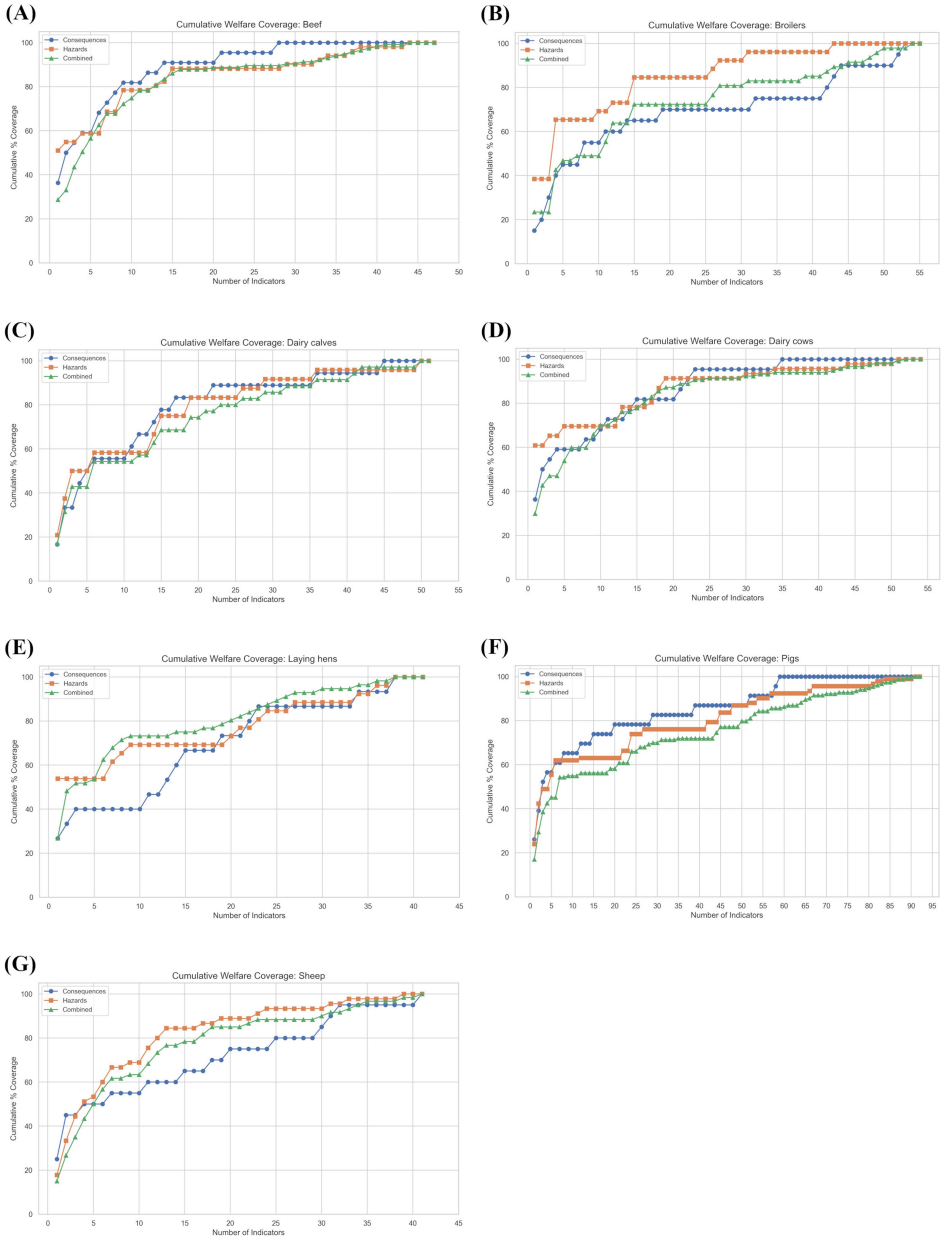
The relationships between the number of indicators combined and cumulative percentage of: a. welfare hazards, b. welfare consequences, and c. welfare hazards x welfare consequences (combination space) explained for: **(A)** Beef cattle, **(B)** Broiler chickens, **(C)** Dairy calves, **(D)** Dairy cows, **(E)** Laying hens, **(F)** Pigs, and **(G)** Sheep. Indicators were added using greedy heuristics.

To investigate this prediction, the relationship between the number of indicators included and the cumulative *Coverage* (expressed as a percentage of the total) achieved was plotted. Three dimensions of *Coverage* were considered: (1) welfare consequences, (2) welfare hazards, and (3) welfare hazards x welfare consequences (‘combination space’) for each species of farm animal (Figures 2A–G). The result showed that for each of the three dimensions, the cumulative percentage *Coverage* exhibited a saturating exponential function as more indicators were combined. Each plot also contained distinctive ‘plateaus’ where the ‘greedy’ addition of the next-best welfare indicator did not add any new *Coverage* to the existing combination.

**Figure 3:**
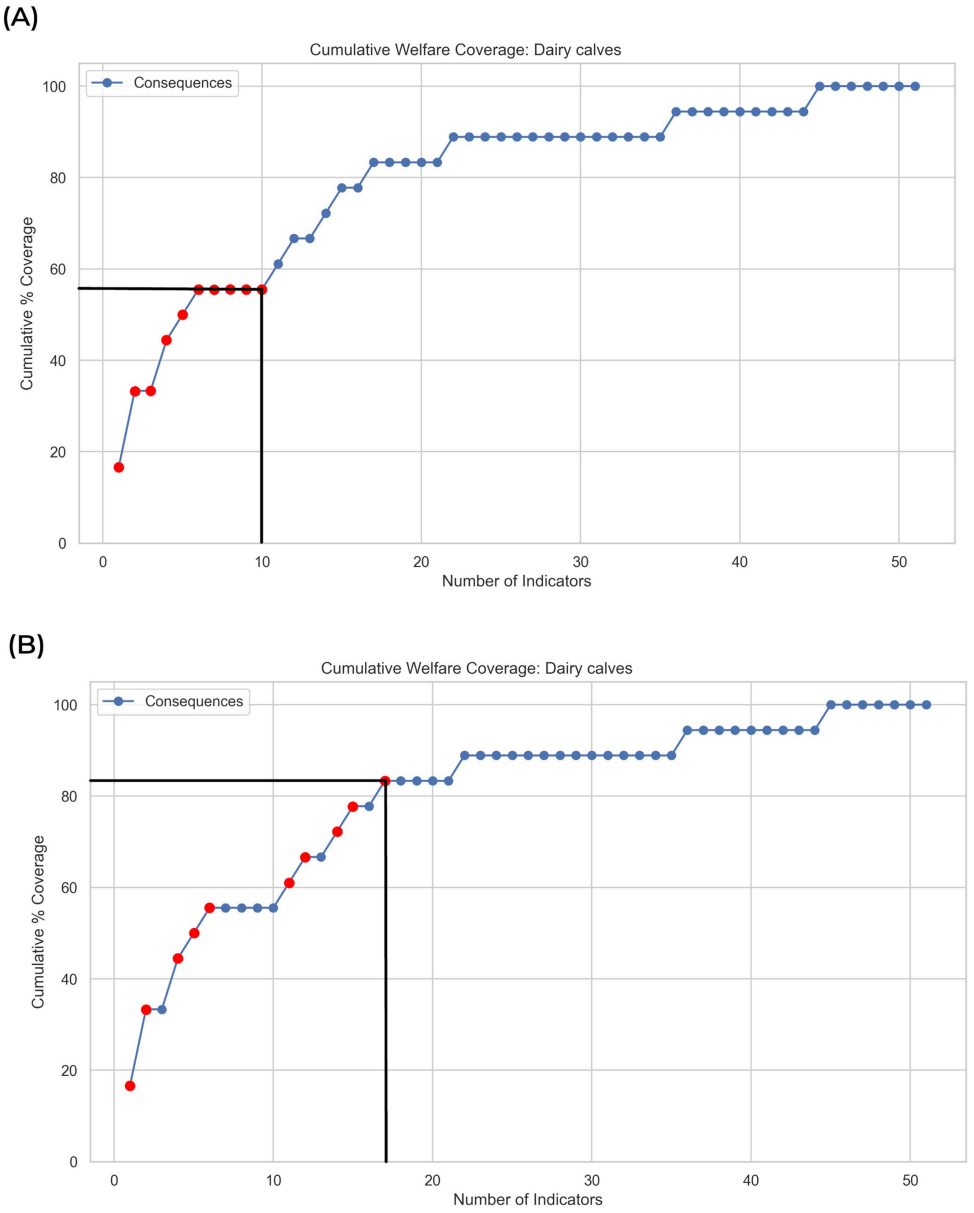
The illustrated use-case only seeks to maximise the number of unique welfare consequences associated with the selection of 10 welfare indicators for dairy calves. **(A)** The greedy algorithm erroneously selects indicators that add no additional coverage (plateaus) whereas **(B)** an algorithm using branch-and-bound methods with backtracking finds the optimum solution.

The existence of ‘plateaus’ in the cumulative plots illustrates where greedy algorithms can select a false optimum. To further elaborate, the case of a user who seeks to find the combination of 10 welfare indicators that maximises the number of associated welfare consequences for dairy calves was considered (Figure 3A). A greedy algorithm would select the top 10 welfare indicators ranked by the number of linked welfare consequences (in this example, explaining approximately 56% of all potential welfare consequences). In contrast, an algorithm using branch-and-bound and backtracking methods would only select the next welfare indicator in the ranked list if it added additional (new) welfare consequences to the solution (in this example, explaining approximately 83% of all potential welfare consequences; Figure 3B). Therefore, to ensure that the final tool developed avoided falsely selecting indicators that did not expand *Coverage*, the project progressed immediately to develop a full algorithm using branch-and-bound methods and backtracking because such algorithms can efficiently identify the true optimum combination.

### 3.4 Enhanced optimisation algorithm using SCIP

#### 3.4.1 Objective function performance

The objective function exhibited a saturating exponential function as the number of selected indicators increased (Supplementary material 1), consistent with diminishing marginal gains in the objective value. In contrast to the stepwise pattern characteristic of the greedy algorithm, the optimisation-based approach yielded a continuous, monotonic increase because indicators were only added when they increased the objective function (i.e., there were no ‘plateaus’).

The shape of this relationship varied by species. For example, the curve for pigs had a higher asymptotic value, resulting from the greater number and diversity of available indicators for this species. The curvature of the function resulted from the relative proportion of ‘broad’ versus ‘narrow’ indicators. A higher proportion of ‘broad’ indicators produced a steeper initial gradient, which is indicative of rapid gains from broadly applicable measures, whereas a predominance of ‘narrow’ indicators led to a more gradual approach to the asymptote due to small incremental improvements from narrowly targeted measures at higher indicator counts.

#### 3.4.2 Solution stability and sensitivity

The composition of the objective function shifted predictably in response to increases in the weighting of *Coverage* (ω_coverage_), with increased contribution from the amplified dimension and a corresponding reduction from others (Supplementary material 2).

These shifts did not destabilise the optimisation process or yield spurious results (e.g., selection collapse or invalid solutions), indicating that the enhanced algorithm tolerated moderate tuning of user-defined priorities without losing solution quality.

#### 3.4.3 Computational efficiency

Tests of computation efficiency were conducted to ensure that the tools would find optimal solutions within a short time limit. For all species of farm animal, it was found that even solutions involving combinations of up to 50 indicators were calculated in less than 200 milliseconds. The early stopping mechanism was not activated in any runs of the algorithm.

Increasing the desired number of indicators in the solution from 5 to 50 (in 10 steps of 5) had no detectible significant effect on the runtime required to obtain a solution for any farm animal species {mean values (ms) across 15 runs: 75.27 [95% CI (74.54, 76.00)] for Beef cattle, 85.97 [95% CI (84.91, 87.03)] for Broilers, 83.27 [95% CI (81.50, 85.03)] for Dairy calves, 87.50 [95% CI (85.49, 89.50)] for Dairy cows, 70.11 [95% CI (68.68, 71.54)] for Laying hens, 132.82 [95% CI (129.97, 135.66)] for Pigs, and 73.04 [95% CI (72.05, 74.04)] for Sheep}.

The longer runtimes observed for pigs arose because of the larger number of indicators identified for this species (92 indicators for pigs vs. a range of 42–54 indicators for the other species).

### 3.5 Case study: broilers

The hypothetical user was a food business that wanted to measure six welfare indicators to demonstrate the year-on-year impact of its management of the welfare of broilers in its supply chain. The sustainability team of the business sought to work on welfare hazards that had the largest impact on bird welfare and that were easy to mitigate. Furthermore, they only wanted to adopt welfare indicators that were easy to use.

#### 3.5.1 Application of the greedy algorithm

The simple greedy algorithm operating on a ranked list of welfare hazard *Coverage* enabled the selection of six welfare indicators that were linked to 17 of the 26 unique welfare hazards (65.4%) and 8 of the 20 unique welfare consequences (40.0%) in *Coverage* space (Supplementary material 3).

However, it was evident that while *injurious pecking* and *bruises* were associated with a relatively large number of welfare hazards and welfare consequences, they did not contribute any new hazards to the cumulative pool if plumage damage were to be selected first.

Consequently, the sustainability team applied the enhanced algorithm to identify the actual optimum (and avoid the selection of duplicate hazards and consequences).

#### 3.5.2 Enhanced algorithm using SCIP

The enhanced algorithm was applied with weighting factors prioritising *Coverage* but also optimising *Ease of indicator use*, and *Impact of welfare consequence*, and *Ease of hazard mitigation*.

The enhanced algorithm selected a different set of six welfare indicators. These were linked to 17 of the 26 unique welfare hazards (65.4%) and 8 of the 20 unique welfare consequences (40.0%) in *Coverage* space (Table 6). While the number of associated welfare hazards and welfare consequences did not change from the greedy solution, the enhanced algorithm substituted two of the originally selected indicators (*injurious pecking* and *bruises*) for alternatives (*wounds* and *hockburn*). These replacements offered equivalent or greater overall utility when all weighted criteria were considered.

**Table 6:** The six welfare indicators for broilers that optimise the combination of: (1) Coverage (welfare hazards and consequences), (2) Impact of welfare consequence, (3) Ease of hazard mitigation, and (4) Ease of indicator use.

Welfare indicator	Hazard coverage (% of total)	Consequence coverage (% total)	Impact of welfare consequence (*n*)	Ease of hazard mitigation (*n*)	Ease of indicator use
Retained from the original greedy algorithm					
Plumage damage	38.5%	15.0%	Low (2) & High (9)	Easy (5), Moderate (4) & Difficult (2)	Easy
Lethargy	30.8%	15.0%	Low (7) & High (2)	Easy (3) & Difficult (6)	Easy
Footpad dermatitis	23.1%	15.0%	High (6)	Easy (2), Moderate (2) & Difficult (2)	Easy
Feather and body dirtiness	19.2%	15.0%	Low (4) & High (1)	Easy (2), Moderate (2) & Difficult (1)	Easy
Removed					
Injurious pecking	34.6%	15.0%	Low (2) & High (9)	Easy (5), Moderate (4) & Difficult (2)	Moderate
Bruises	34.6%	5.0%	High (9)	Easy (4), Moderate (3) & Difficult (2)	Moderate
Added					
Wounds	19.2%	10.0%	High (7)	Easy (4), Moderate (1) & Difficult (2)	Easy
Hockburn	15.4%	10.0%	High (4)	Easy (2), Moderate (1) & Difficult (1)	Easy

Weighting factors were set to favour coverage and equally balance the other three factors in the decision set (ω_coverage_ = 5 (α. HazardCoverage = 0.5 + β. ConsequenceCoverage = 0.5), ω_easiness_ = 1, ω_impact_ = 1 and ω_mitigation_ = 1). The welfare indicators listed under ‘Removed’ are excluded from the greedy solution and are replaced by those listed under ‘Added’.

#### 3.5.3 Computational efficiency

The mean computation time for the enhanced algorithm remained consistently low for all combinations of indicators evaluated. There was no detectible significant effect on the runtime to obtain a solution as the desired number of indicators was increased from 5 to 50 [*F*_(9,140)_ = 0.7061; NS]. The mean runtime was 91.71 ms [95% CI (89.85, 93.57)]. Although there was some variability, especially at lower indicator numbers, the 95% confidence intervals were narrow and overlapped, indicating no significant change in compute time. The linear trend line had a slight positive gradient, but the slope was minimal, and the confidence band was narrow, suggesting that the algorithm scaled efficiently with increasing problem size (Supplementary material 4).

#### 3.5.4 Scenario analysis

**Figure 4:**
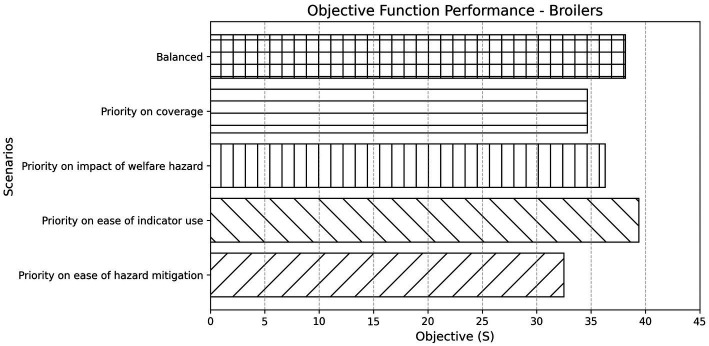
Objective function performance across different weighting scenarios for broilers. Bars represent the final objective score (S) achieved under each scenario, reflecting the combined value of selected indicators based on their contribution to coverage, impact, and feasibility dimensions. The “Balanced” scenario applies equal weighting to all criteria, while the others apply increased weighting to a specific aspect of interest.

For broilers, the degree to which the elements of the objective function were weighted was important. For example, was it more important to maximise the breadth of hazard and consequence *Coverage*, or to favour indicators that were easier to implement on-farm and target welfare issues that are both impactful and readily mitigated? Adjusting these weights altered the optimisation landscape and shifted the selected indicator set significantly (Figure 4).

#### 3.5.5 Robustness testing

Substantial perturbations in weighting factors did not result in any major changes in the selection of welfare indicators for broilers (Supplemental material 4). This suggests that the solution space is stable across a range of input preferences, and that small or even moderate deviations in how indicators are weighted do not result in radically different outcomes. This provides confidence in the reliability and predictive consistency of the model.

### 3.6 Case study: growing/finishing pigs

The user is an animal welfare certification scheme provider that wants to measure six welfare indicators to demonstrate the welfare status of growing and finishing pigs across third-party certified farms. Their goal is to ensure that selected indicators are both scientifically robust and feasible to implement during periodic assessments. Specifically, they seek indicators that are able to cover a broad range of welfare hazards and consequences; address high-impact risks to animal welfare; are practical to assess during on-farm audits and focus on hazards that are realistically mitigable.

#### 3.6.1 Application of the greedy algorithm

A simple greedy algorithm operating on a ranked list of welfare hazard *Coverage* enabled the selection of six welfare indicators that were linked to 27 of the 58 unique welfare hazards (46.6%) and 7 of the 16 unique welfare consequences (43.8%) in *Coverage* space (Supplementary material 5).

#### 3.6.2 Enhanced algorithm

In contrast to the broiler case study, the enhanced optimisation method using branch-and-bound with backtracking produced the same indicator set, confirming that the greedy solution was, in this case, also globally optimal given the input criteria and weights.

The algorithm suggested the selection of six welfare indicators that were also linked to 27 of the 58 unique welfare hazards (46.6%) and 7 of the 16 unique welfare consequences (43.8%) in *Coverage* space (Table 7). While these indicators were the same as the set determined by the greedy algorithm (i.e., none were added or removed), the use of the enhanced algorithm still added value because it validated the result under a multi-dimensional objective function and provided insight into why each indicator was selected as it also quantified its contribution to *Coverage*, *Ease of indicator use*, *Impact of welfare consequence*, and *Ease of hazard mitigation*.

**Table 7:** The six welfare indicators that optimise the combination of: (1) Coverage, (2) Impact of welfare consequence, (3) Ease of hazard mitigation, and (4) Ease of indicator use for meat pigs.

Welfare indicator	Hazard coverage	Consequence coverage	Impact of welfare consequence	Ease of hazard mitigation	Ease of indicator use
Retained from the original greedy algorithm					
Calluses and bursitis (pressure injuries)	31.0%	25.0%	High (22)	Easy (7), Moderate (11) & Difficult (4)	Easy
Body condition	24.1%	12.5%	Low (9) & High (5)	Easy (6), Moderate (7) & Difficult (1)	Moderate
Ear lesions	22.4%	12.5%	Low (1) & High (13)	Easy (6), Moderate (7) & Difficult (1)	Easy
Tail lesions	22.4%	12.5%	Low (1) & High (13)	Easy (6), Moderate (7) & Difficult (1)	Easy
Body lesions	20.7%	6.3%	High (12)	Easy (4), Moderate (7) & Difficult (1)	Easy
Leg injuries	20.7%	6.3%	High (14)	Easy (5), Moderate (6) & Difficult (1)	Easy

Weighting factors were set to favour coverage and equally balance the other three factors in the decision set (ω_coverage_ = 5 (α. HazardCoverage = 0.5 + β. ConsequenceCoverage = 0.5), ω_easiness_ = 1, ω_impact_ = 1 and ω_mitigation_ = 1). No welfare indicators were added or removed from the greedy solution.

Furthermore, analysis of the solution space revealed that each selected indicator contributed uniquely to the objective function. No indicator was redundant, and no alternative set achieved a higher composite score under the defined constraints. This reinforced confidence in the selection and demonstrated the algorithm’s robustness for applications where audit efficiency, *Coverage*, and welfare relevance must all be balanced.

#### 3.6.3 Computational efficiency

The mean computation time for the enhanced algorithm remained consistently low for all combinations of indicators evaluated. There was no detectible significant effect on the runtime to obtain a solution as the desired number of indicators was increased from 5 to 50 [*F*_(9,140)_ = 0.7648; NS]. The mean runtime was 82.51 ms [95% CI (81.34, 83.69)]. The linear trend is flat, with an almost negligible slope and a tightly bounded confidence band, confirming that computational load does not appreciably increase with problem size. Compared to broilers, the computation times for meat pigs are slightly more consistent and show less variability across indicator counts, suggesting even more uniform performance of the algorithm in this context (Supplementary material 6).

#### 3.6.4 Scenario analysis

**Figure 5:**
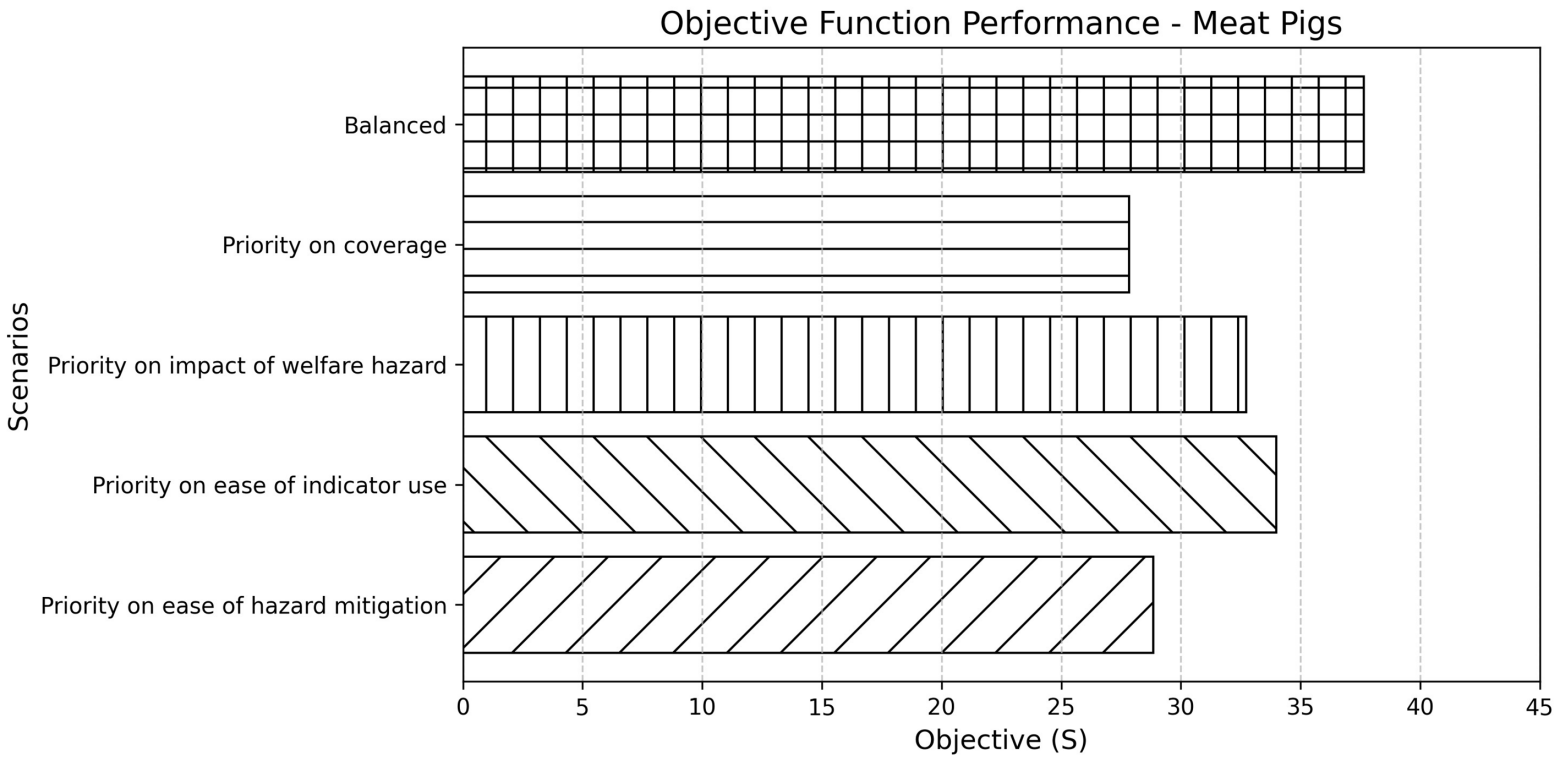
Objective function performance across different weighting scenarios for meat pigs. Bars represent the final objective score (S) achieved under each scenario, reflecting the combined value of selected indicators based on their contribution to coverage, impact, and feasibility dimensions. The “Balanced” scenario applies equal weighting to all criteria, while the others apply increased weighting to a specific aspect of interest.

For meat pigs, the degree to which the elements of the objective function were weighted was again important. Adjusting these weights altered the optimisation landscape and modified the selected indicator set significantly (Figure 5). The “Priority on ease of indicator use” scenario produced the highest objective score, whereas “Priority on ease of hazard mitigation” resulted in the lowest, indicating trade-offs between dimensions when emphasising specific priorities.

#### 3.6.5 Robustness testing

Substantial perturbations in weighting factors did not result in any major changes in the selection of welfare indicators for meat pigs (Supplementary material 6). This again suggested that the solution space was stable across a range of input preferences, and that small or even moderate deviations in how indicators are weighted did not result in radically different outcomes. This provided further confidence in the reliability and predictive consistency of the algorithm’s output.

This case study illustrated how the optimisation framework can support certification bodies in defining concise, evidence-based indicator sets that are aligned with both scientific principles and operational feasibility.

## 4 Discussion

The study presented in this paper aimed to develop and compare tools to discover optimal combinations of welfare indicators using multi-criteria decision analysis (MCDA). Two algorithms were developed: (a) a simple greedy algorithm, (b) an enhancement of the greedy algorithm using SCIP, which identified the global optimum using branch-and-bound methods and backtracking. Both algorithms were evaluated using a database that was populated with information from the European Food Safety Authority AHAW panel’s risk-based assessments of animal welfare and other published literature for multiple species of farm animals. The ultimate objective was to enable users to select combinations of welfare indicators that are both effective in detecting the most serious welfare hazards, measure their consequences, and mitigate the impact of such consequences using indicators that are straightforward to implement in real-world monitoring programmes.

### 4.1 Moving beyond iceberg indicators

The approaches developed in the present study build on the concept of iceberg indicators because of their ability to serve as proxy measures for multiple underlying welfare consequences and welfare hazards. The *Coverage* construct was used to quantify the number of welfare hazards and/or welfare consequences that were associated with each indicator. Consistent with the concept of iceberg indicators, it was found that some indicators were linked to only a few welfare hazards and/or consequences (i.e., were ‘narrow’ in *Coverage* and had a low level of ‘icebergyness’), whereas others were linked to more (i.e., were ‘broad’ in *Coverage* and had a higher level of ‘icebergyness’). This is consistent with EFSA’s methodological guidance for developing welfare opinions, where the term specificity of an ABM refers to its ability to identify animals that are not experiencing a particular welfare consequence and, by extension, those that are (46). In this context, iceberg indicators can be viewed as being analogous to ‘broad’ indicators. It is noted, however, that without reference to metadata such as sensitivity the use of *Coverage* alone may lead to the omission of ‘narrow’ indicators that are linked to a welfare consequence that has a large impact on the welfare of the animals.

It was found that some indicators shared a large number of common welfare hazards and welfare consequences. This observation is not new. The Welfare Quality® project commented that some measures may be linked to several welfare dimensions (47). To make an overall assessment of animal welfare, they proposed to select measures (indicators) using weighted sums and comparison with minimal requirements and further evaluation of the precision with which they could be deployed. In later studies, attempts to reduce the number of indicators to focus on certain iceberg indicators for welfare assessment explored techniques such as partial least square structural equation modelling, where measurable indicators that explain the highest variance in the latent variables (e.g., animal welfare) are included in the models (48). The present research is also based on the assertion that welfare indicators share *Coverage* to varying degrees. However, the ways in which indicators with common (shared) welfare hazards and welfare consequences are treated are managed differently by the two algorithms. When operating only on *Coverage*, the greedy algorithm merely selects indicators in ranked order and, in doing so, it potentially suggests combinations that have overlapping welfare hazards and/or welfare consequences (i.e., it does not account for marginal gains in *Coverage*). In contrast, the enhanced algorithm only adds a welfare indicator to the solution when there is a demonstrable increase in the full objective function, thereby avoiding duplications in *Coverage*.

During the development of the greedy algorithm, the notion of ‘combination space’ was explored briefly as both a conceptual and computational construct. This construct mapped the unique pairings of welfare hazards and consequences linked to each indicator. In plots of cumulative *Coverage* of ‘combination space’ using the greedy algorithm, greater resolution was observed than in approaches that consider hazards or consequences independently (fewer plateaus arose from duplications in *Coverage*). Analysis within ‘combination space’ makes it possible to assess each indicator’s unique contribution, including whether it captures novel hazard-consequence associations that would otherwise remain undetected. This possibility was not pursued further within the present exercise, as the goal of the enhanced algorithm was to enable welfare hazards and consequences to be weighted differentially by the user. However, the selection of combinations of indicators within ‘combination space’ using the enhanced algorithm remains potentially worthy of further investigation. In doing so, the method could enable finer differentiation between indicators and provide a more accurate reflection of the integrated risk model proposed by EFSA, which considers both the nature of the hazard and the most relevant welfare consequences for each species.

### 4.2 Method development

The proof-of-concept study applied to datasets for broiler chickens and laying hens demonstrated that small, strategically chosen subsets of indicators could match or exceed the explanatory power of much larger combinations of indicators. This outcome has practical significance, suggesting that welfare monitoring protocols can be streamlined without compromising scientific integrity. This could be especially valuable in resource-constrained settings. It should be noted however, that the definition of success will depend on the desired/target level of *Coverage*.

While the greedy algorithm was fast and transparent in its operation, it was prone to include indicators that added no additional explanatory power to the existing combination. In contrast, the enhanced algorithm permits users to prioritise attributes such as *Coverage*, *Ease of indicator use*, *Impact of welfare consequence*, and *Ease of hazard mitigation* independently. This enabled the tailoring of indicator selection to specific use-cases. As these weights could be ‘tuned’ by the user, the algorithm did not impose a fixed hierarchy. This could allow decision-makers to explore trade-offs and impose different priorities in the optimisation process. For example, it is well known that some indicators require a significant amount of time to measure. In practice, this may make them unfeasible to use within a time-bound farm visit (49) and the ability to adapt priorities will help to reconcile such trade-offs between scientific precision and practical feasibility. For example, indicators offering broad levels of *Coverage* may be expensive or complex to apply routinely, while simpler indicators may fail to capture important welfare hazards and welfare consequences.

The broiler chicken case study provided a clear illustration of this issue. The greedy algorithm selected indicators with strong individual *Coverage* but limited incremental value, as many covered overlapping risks. In contrast, the enhanced algorithm selected sets that replaced redundant indicators with those offering better trade-offs between *Coverage*, *Ease of indicator use*, *Impact of welfare consequence*, and *Ease of hazard mitigation* potential. This supports the increasing recognition that multi-criteria decision analysis (MCDA) could be a valuable tool in animal welfare science, as highlighted in recent literature advocating for structured decision support in ethically and logistically complex contexts (50–52).

### 4.3 Determining weighting values

Selecting appropriate weights remains a complex task. The present paper highlights similarities with weighting challenges experienced in other sectors employing MCDA tools (53). Without systematic methods for weight assignment, such as stakeholder elicitation or performance-based calibration, there is a risk that choices may appear arbitrary. For real-world implementation, it may be necessary to engage stakeholders in structured processes to derive weightings in a transparent and reproducible way. In doing so, it must be acknowledged that stakeholders may assign different weighting values for their particular use-case. For example, the competent authority in an individual Member State of the European Union may weight ease of indicator use differently to the policymakers who formulate the underlying legislation against which farming practices are regulated. Further dialogue and research may be necessary to define a suitable process for weighting the selection of welfare indicators. However, several methods have been documented in relation to human healthcare which may provide further insights when developing a process. A review can be found in a report published by the MCDA Emerging Good Practices Task Force of the International Society for Pharmacoeconomics and Outcomes Research (54).

In this study, several hypothetical weighting scenarios were evaluated to model different decision-making contexts, including *Best-case*, *Severity-* and *Mitigation-focused* strategies. These were useful for demonstrating the sensitivity of the optimisation output to changes in user priorities. While the choice of weights influenced which indicators were selected, the number of indicators requested proved to be the most significant driver of overall *Coverage* which increased rapidly with the first few indicators, but then slowly plateaued. This implies that adding indicators beyond a certain number offer diminishing returns in terms of added welfare information.

While univariate analyses illustrate which single criteria are most influential, examining one weight at a time masks interactive effects that may exist between selection criteria (e.g., *Coverage* × *Ease of use*). For example, simultaneously increasing ω_coverage_ and decreasing ω_easiness_ could lead to quite different outcomes compared with when altering a single weight in isolation. As a result, univariate assessments may underestimate instability if the objective function only shows sensitivity when multiple weights are perturbed together.

Multivariate analyses could provide a more realistic assessment of stability. Decision making rarely involves modifying the weight of a single selection criterion in isolation. The exploration of combined weight changes would allow for the identification of threshold effects or nonlinearities that may not be apparent with univariate approaches. For example, a set of indicators might remain stable under adjustments to individual weights but alter substantially when two or more weights are perturbed together. Such behavior signals potential instability in the optimisation, and small but coordinated changes in stakeholder priorities could yield markedly different solutions. Recognising and quantifying these regions of instability is important, both for gauging the reliability of the indicator set and for ensuring that the optimisation is not unduly sensitive to subjective weight assignments.

Although a full multivariate sensitivity analysis lies outside of the scope of the present study, the univariate analyses reported offers a first step to illustrate how shifts in weighting values affect outcomes of the optimisation. Future work should extend this approach to explore nonlinear interactions and robustness under simultaneous perturbations of multiple weights.

### 4.4 Assessing performance

To assess performance, results from the greedy algorithm were compared with those from the enhanced algorithm. In many cases, the greedy method plateaued early because the most informative indicators were selected first. Thereafter, newly added indicators failed to expand *Coverage* due to redundancy. This behavior revealed the non-additive nature of information across indicators and highlighted inherent inefficiencies in naïve selection strategies. The enhanced algorithm was better able to consider duplication in *Coverage* between indicators to overcome this limitation. It was able to identify indicator sets where the combined contribution was maximised, even when individual indicators had modest standalone scores. Crucially, this improved accuracy was not associated with a large increase in computational overhead. All optimisation tasks were completed in less than 0.2 s, even with real-world data, suggesting that the method is suitable for immediate deployment in interactive decision support tools.

While the enhanced algorithm demonstrated strong computational performance for the current dataset, further work is required to explore how runtime will scale with larger or more complex sets of indicators and selection criteria (e.g., creating trade-off curves for runtime vs. complexity). It is likely that runtimes will not be directly proportional to complexity because of the potential for ‘computational thresholds’ where compute time could rise disproportionately. Investigating such scaling effects is important if the approach is to be applied to larger databases or refined by incorporating more selection criteria. Furthermore, performance is also of relevance to decision-making because the algorithms will require alignment with the priorities of different stakeholder groups (such as veterinarians, auditors, or policymakers). The degree to which algorithms respond to this diversity is therefore an important dimension of performance in its own right. Systematic scenario testing can help ensure that results remain credible and interpretable across multiple stakeholder use-cases. More in-depth scenario testing would also help to further refine the algorithms (where necessary) and support the deployment of the method in practice.

### 4.5 Strengths and weaknesses of the approach(es)

A key strength of the enhanced algorithm is its flexibility to balance the evidence-based categorisation of welfare indicators with their operational feasibility. A further strength is its ability to generate stable solutions across a wide range of use-cases. This stability is valuable, as it enhances trust in the optimisation results and reduces the likelihood that small variations in stakeholder priorities will produce radically different outputs. However, excessive stability could be a weakness if it prevents the optimisation from capturing the expected diversity in perspectives. Finding an appropriate balance between robustness and flexibility remains a key consideration for future applications, particularly in participatory settings where stakeholders may wish to explore how their priorities translate into different indicator sets.

In the present paper, certain indicators continue to be selected regardless of substantial changes in weighting. This may imply that these indicators offer intrinsically high utility across multidimensions and either reflect strong underlying linkages to key welfare hazards and consequences, or a combination of feasibility and explanatory power that makes them consistently optimal. However, the limited impact of weight perturbations may also reflect redundancy among candidate indicators, where several alternatives offer similar levels of utility. In such cases, the enhanced algorithm may converge on a subset of equally acceptable solutions that differ little in *Coverage* or score, even under varying conditions (as observed in the broiler chicken proof-of-concept study).

If optimisation outcomes are not overly sensitive to changes in the weighting inputs, this can facilitate consensus-building among stakeholders with differing priorities. It suggests that diverse viewpoints may still lead to convergent solutions, supporting broader adoption of welfare monitoring tools. Moreover, it implies that implementation decisions can be made with less risk of error from minor misjudgements or variations in assigned priorities. Alternatively, the absence of observable differences may indicate limited sensitivity in the weighting system, especially if weights are applied to ordinal metadata (e.g., ‘Easy’, ‘Moderate’, ‘Difficult’). In such cases, large changes in weight values may still result in minimal shifts in the objective function because of discrete scoring steps. This points to a methodological limitation, where finer granularity in input data or a more continuous scoring system might be needed to detect more subtle trade-offs.

The approach aligns with discussions about the trade-offs between accuracy and ability to implement welfare indicators in animal welfare science (1). For instance, high-*Coverage* indicators may be too complex or costly to assess routinely, while easier-to-use indicators may miss critical welfare dimensions. However, one notable constraint in this study was the use of categorical metadata to describe attributes such as *Ease of indicator use* or *Ease of hazard mitigation*. These were expressed using ordinal scales (such as Easy, Moderate or Difficult), which, while easy to interpret, reduce the resolution of the objective function. In practice, this meant that many indicators received the same weighted contribution and became indistinguishable in optimisation outputs. This often resulted in the identification of multiple equivalently optimal indicator sets, where several combinations achieved the same overall score.

While this ambiguity does not reduce the total welfare information captured, it highlights a methodological limitation. Specifically, ordinal data can reduce the discriminatory power of the model, which may in turn limit the precision of the final output. Future applications would benefit from a more standardised scoring system across expert panels or finer-grained input values derived from empirical evidence. The use of visual analogue scales also holds great potential in this regard, as they generate continuous data and provide an objective means of identifying disagreement between experts during an elicitation exercise (e.g., when the inter-expert variability exceeds a predefined threshold).

Another issue concerns the interpretability of the output. Sensitivity and scenario analyses may help to improve transparency by revealing how the selection of indicators depends on the weighting of the selection criteria. However, if too many use-cases are investigated, the resulting complexity risks overwhelming end-users who may not be familiar with optimisation methods. The challenge is to ensure that the analysis remains sufficiently transparent to inspire confidence, while avoiding excessive technical detail that could mask the main messages for decision-makers. Clear communication of optimisation outcomes will be of central importance. One strategy is to use ‘envelopes’, where results are reported as the proportion of scenarios in which a given indicator is selected (e.g., “Indicator X appeared in >80% of runs”). This communication strategy avoids the presentation of multiple optimal solutions and presents a single measure of consistency across potential scenarios. This may help to strengthen stakeholder confidence, facilitate policy uptake, and provide a more intuitive picture of where consensus is likely to emerge.

The algorithms could also be criticised in that they assume welfare hazards are mutually exclusive and do not interact. This may not be the case. Different hazards may jointly lead to more severe welfare consequences, as may be the case with a multifactorial problem such as tail biting in pigs (58). Further research is required to better understand such interactions as they are likely to differ on a case-by-case basis.

### 4.6 Next, steps

The approaches outlined in the present paper are interoperable in that they are data agnostic. While the dataset used was based on the EFSA scientific opinions and other published information, the algorithms can be applied easily to other datasets. It should also be noted that the results presented are derived from data applicable to Norwegian farming systems, which differ in some respects from more conventional European systems (the use of sow farrowing crates is not allowed, and tie stalls are still commonly used in cattle housing). However, the expansion of the dataset to encompass other European or global farming systems remains an important next step. The EFSA scientific opinions predominantly reflect farming systems and priorities within the European Union and can be applied (to some extent) to systems of production in other regions (e.g., North America). As such, the indicators and their relative importance may not be directly transferable to other geographies where production systems, resource availability, and societal expectations differ. It must also be recognised that societal values and the scientific understanding of animal welfare evolve over time. Therefore, weighting and the relevance of particular indicators may shift. This underscores the importance of viewing optimisation not as a one-off solution, but as a dynamic tool that can be updated as knowledge, stakeholder priorities, and welfare expectations develop. Far from being a weakness, this adaptability may help ensure that welfare assessment frameworks remain aligned with both emerging science and shifting societal values.

The enhanced algorithm can be further developed in several ways. First, the approach has not yet been extended into ‘combination space’ to consider the varying associations that exist between welfare hazards and welfare consequences (8). Second, animal welfare science currently does not yet contribute much information on positive welfare (55). As this field develops, the objective function could be adapted to incorporate metrics with positive valence (e.g., indicators associated with positive welfare consequences).

## 5 Conclusion

In conclusion, this work provides a novel and flexible optimisation framework for selecting animal welfare indicators that balances scientific rigour with operational feasibility. By formalising the notion of *Coverage* and enabling user-defined prioritisation, the method supports more targeted, efficient, and context-appropriate monitoring strategies across species and production systems. The ability to identify small, high *Coverage* indicator sets has the potential to reduce resource demands while maintaining diagnostic power, thereby enhancing the scalability and adoption of welfare assessment programmes. As such, the approach offers practical value to industry, regulators, and researchers aiming to implement welfare monitoring systems that are both evidence-based and adaptable to real-world constraints. The findings reinforce the value of iceberg indicators, demonstrate the analytical advantages of welfare hazard and consequence *Coverage*, and point to the potential of optimisation frameworks to advance the practical implementation of welfare assessment in animal production systems, supporting the quantitative risk assessment on animal welfare.

## Data Availability

The Python code generated for this study can be found in the Mentis SA welfare indicators repository on GitHub (https://github.com/mentis-dev/welfare_indicators).
