# Supplementary material for: Optimising the selection of welfare indicators in farm animals
Source: Front Vet Sci. 2025 Oct 28;12:1661470. doi: 10.3389/fvets.2025.1661470 (PMC12604357; doi:10.3389/fvets.2025.1661470)
Supplement: Supplementary file 1 [file Supplementary_file_1.docx]

Supplementary Material 1

| 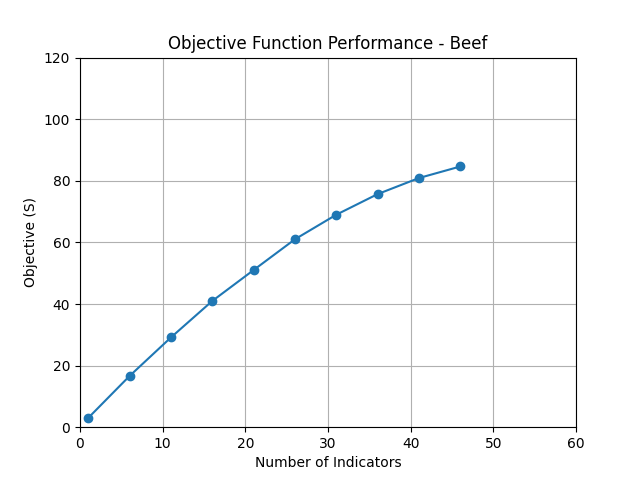 | 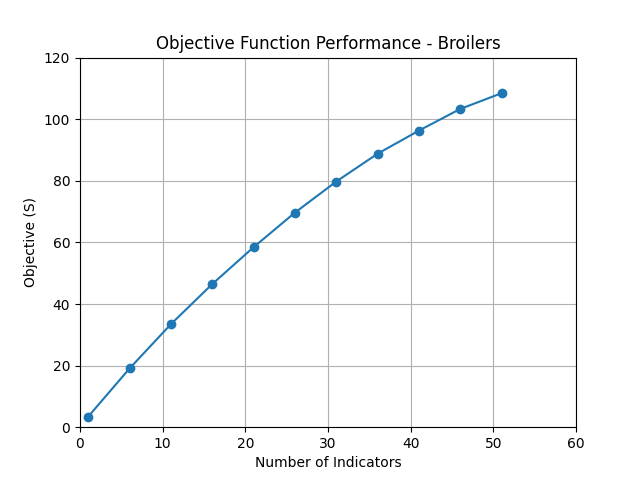 | 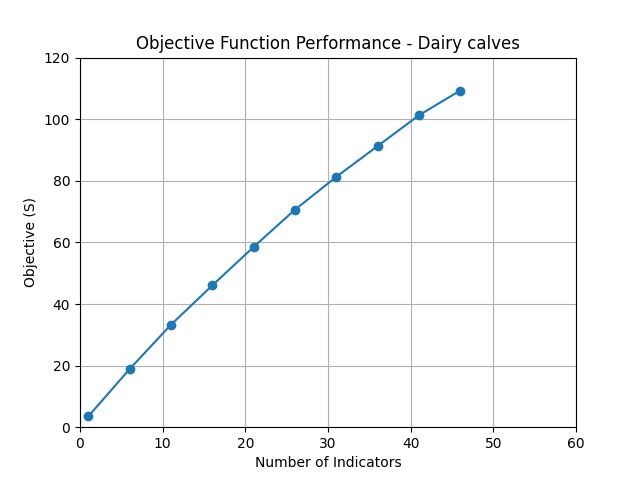 |
| --- | --- | --- |
| 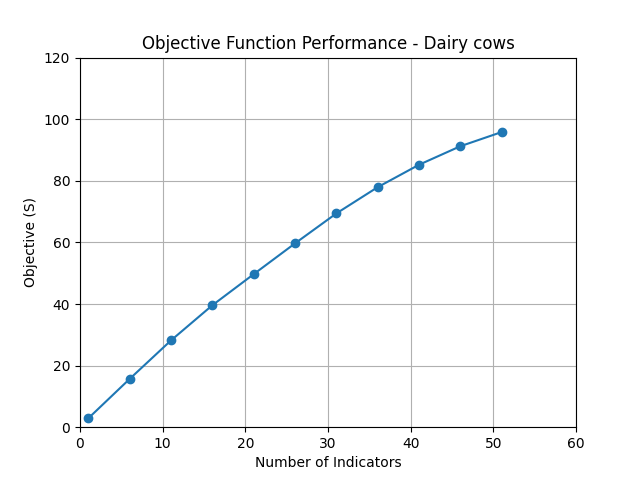 | 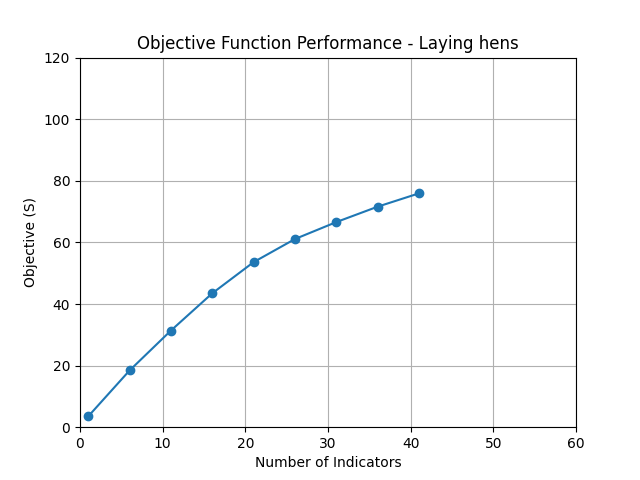 | 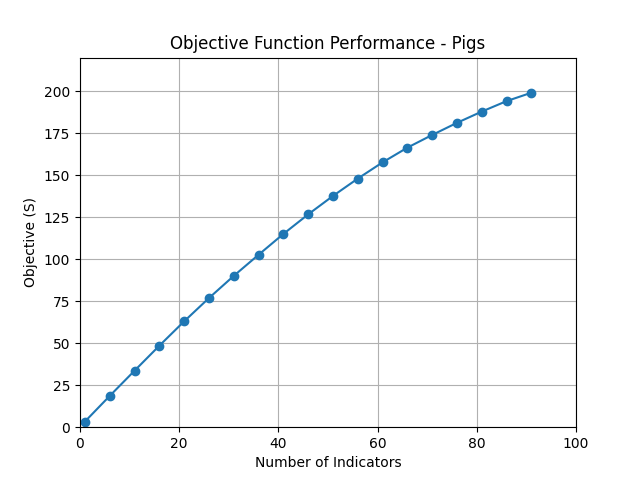 |
| 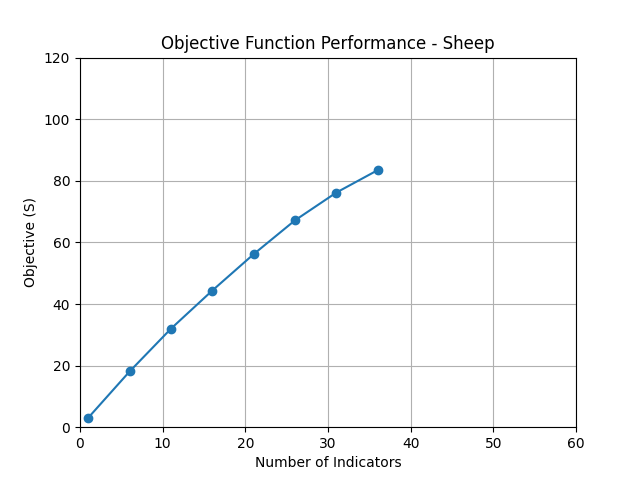 | **Figure 4:** *Relationship between the desired (maximum) number of indicators in the solution (x-axis) and the value of the objective function (S) for:* ***(A)*** *Beef cattle,* ***(B)*** *Broiler chickens,* ***(C)*** *Dairy calves,* ***(D)*** *Dairy cows,* ***(E)*** *Laying hens,* ***(F)*** *Pigs, and* ***(G)*** *Sheep. The optimisation was run using the objective function outlined in Eqns. 8 & 9. Each weighting factor was given equal importance (i.e. coverage weight = 1.0, ease of indicator use weight =1.0, impact of welfare consequence weight = 1.0 and ease of hazard mitigation weight = 1.0), and the relative importance of the number of welfare hazards to the number of welfare consequences was a ratio of 1:1.* | |
