# Supplementary material for: Optimising the selection of welfare indicators in farm animals
Source: Front Vet Sci. 2025 Oct 28;12:1661470. doi: 10.3389/fvets.2025.1661470 (PMC12604357; doi:10.3389/fvets.2025.1661470)
Supplement: Supplementary file 3 [file Supplementary_file_3.docx]

Supplementary Material 3

**Supplementary information 3:** *The top six welfare indicators for broilers ranked first by the number of welfare hazards (H) and then by the number of welfare consequences (C). The greedy algorithm adds welfare indicators according to their rank order {1,…,6}. Shaded cells are welfare hazards and welfare consequences that are unique to the growing indicator set, unshaded cells are welfare hazards and welfare consequences that already exist in the growing indicator set.*

| **Rank** | **1** | | **2** | | **3** | | **4** | | **5** | | **6** | |
| --- | --- | --- | --- | --- | --- | --- | --- | --- | --- | --- | --- | --- |
|  | **Plumage damage** | | **Injurious pecking** | | **Bruises** | | **Lethargy** | | **Footpad dermatitis** | | **Feather and body dirtiness** | |
|  | *H* | *C* | *H* | *C* | *H* | *C* | *H* | *C* | *H* | *C* | *H* | *C* |
| ***Coverage (n)*** | 10 | 3 | 10 | 3 | 9 | 1 | 8 | 3 | 6 | 3 | 5 | 3 |
| ***Total (n)*** | 26 | 20 | 26 | 20 | 26 | 20 | 26 | 20 | 26 | 20 | 26 | 20 |
| **% Total** | 38.5% | 15.0% | 38.5% | 15.0% | 34.6% | 5.0% | 30.8% | 15.0% | 23.1% | 15.0% | 19.2% | 15.0% |
| **Added to solution (n)** | - | - | 0 | 0 | 0 | 0 | 7 | 3 | 0 | 1 | 0 | 1 |
| **Cumulative (n)** | 10 | 3 | 10 | 3 | 10 | 3 | 17 | 6 | 17 | 7 | 17 | 8 |
| **Cumulative (%)** | 38.5% | 15.0% | 38.5% | 15.0% | 38.5% | 15.0% | 65.4% | 30.0% | 65.4% | 35.0% | 65.4% | 40.0% |
| 1 | Crusted litter | Group (social) stress | Crusted litter | Group (social) stress | Crusted litter | Soft tissue lesions and integument damage | High environmental temperature | Cold stress | High stocking density | Gastro-enteric disorders | High stocking density | Gastro-enteric disorders |
| 2 | High light intensity | Inability to perform exploratory or foraging behaviour | High light intensity | Inability to perform exploratory or foraging behaviour | High light intensity |  | High or low humidity | Gastro-enteric disorders | Poor housing design and allocation of resources | Restriction of movement | Inadequate environment | Inability to perform comfort behaviour |
| 3 | High stocking density | Soft tissue lesions and integument damage | High stocking density | Soft tissue lesions and integument damage | High stocking density |  | High stocking density | Heat stress | Poor quality diet | Soft tissue lesions and integument damage | Poor housing design and allocation of resources | Restriction of movement |
| 4 | Inadequate environment |  | Inadequate environment |  | Inappropriate environmental enrichment |  | Low environmental temperature |  | Poor water quality |  | Poor quality diet |  |
| 5 | Inappropriate environmental enrichment |  | Inappropriate environmental enrichment |  | Lack of space |  | Poor quality diet |  | Unbalanced body conformation |  | Poor water quality |  |
| 6 | Lack of space |  | Lack of space |  | Low light intensity |  | Poor ventilation |  | Wet litter |  |  |  |
| 7 | Low light intensity |  | Low light intensity |  | Poor housing design and allocation of resources |  | Poor water quality |  |  |  |  |  |
| 8 | Poor housing design and allocation of resources |  | Poor housing design and allocation of resources |  | Unbalanced body conformation |  | Water restriction |  |  |  |  |  |
| 9 | Unbalanced body conformation |  | Unbalanced body conformation |  | Wet litter |  |  |  |  |  |  |  |
| 10 | Wet litter |  | Wet litter |  |  |  |  |  |  |  |  |  |
