# Supplementary material for: Optimising the selection of welfare indicators in farm animals
Source: Front Vet Sci. 2025 Oct 28;12:1661470. doi: 10.3389/fvets.2025.1661470 (PMC12604357; doi:10.3389/fvets.2025.1661470)
Supplement: Supplementary file 5 [file Supplementary_file_5.docx]

Supplementary Material 5

**Supplementary material 5:** *The top six welfare indicators for meat pigs ranked by the number of welfare hazards (H) and welfare consequences (C). The greedy algorithm adds welfare indicators according to their rank order {1 … 6}. Shaded cells are welfare hazards and welfare consequences that are unique to the growing indicator set, unshaded cells are welfare hazards and welfare consequences that already exist in the growing indicator set.*

| **Rank** | **1** | | **2** | | **3** | | **4** | | **5** | | **6** | |
| --- | --- | --- | --- | --- | --- | --- | --- | --- | --- | --- | --- | --- |
|  | **Calluses and bursitis (pressure injuries)** | | **Body condition** | | **Ear lesions** | | **Tail lesions** | | **Body lesions** | | **Leg injuries** | |
|  | *H* | *C* | *H* | *C* | *H* | *C* | *H* | *C* | *H* | *C* | *H* | *C* |
| ***Coverage (n)*** | 18 | 4 | 14 | 2 | 13 | 3 | 13 | 2 | 12 | 1 | 12 | 1 |
| ***Total (n)*** | 58 | 16 | 58 | 16 | 58 | 16 | 58 | 16 | 58 | 16 | 58 | 16 |
| **% Total** | 31.0% | 25.0% | 24.1% | 12.5% | 22.4% | 12.5% | 22.4% | 12.5% | 20.7% | 6.3% | 20.7% | 6.3% |
| **Added to solution (n)** | - | - | 6 | 2 | 1 | 1 | 1 | 0 | 1 | 0 | 0 | 0 |
| **Cumulative (n)** | 18 | 4 | 24 | 6 | 25 | 7 | 26 | 7 | 27 | 7 | 27 | 7 |
| **Cumulative (%)** | 31.0% | 25.0% | 41.4% | 37.5% | 43.1% | 43.8% | 44.8% | 43.8% | 46.6% | 43.8% | 46.6% | 43.8% |
| 1 | Aggression during the mixing of unfamiliar animals | Locomotor disorders (including lameness) | Aggression during the mixing of unfamiliar animals | Gastro-enteric disorders | Aggression during the mixing of unfamiliar animals | Inability to perform exploratory or foraging behaviour | Competition/aggressive interactions during feeding | Inability to perform exploratory or foraging behaviour | Aggression during mixing of unfamiliar animals | Soft tissue lesions and integument damage | Aggression during mixing of unfamiliar animals | Soft tissue lesions and integument damage |
| 2 | Competition/aggressive interactions during feeding | Resting problems | Competition/aggressive interactions during feeding | Group (social) stress | Competition/aggressive interactions during feeding | Soft tissue lesions and integument damage | Complications of tail docking | Soft tissue lesions and integument damage | Competition/aggressive interactions during feeding |  | Competition/aggressive interactions during feeding |  |
| 3 | Damaging behaviour to conspecifics | Restriction of movement | Grouping of pigs of different age and/or size |  | Damaging behaviour to conspecifics |  | Damaging behaviour to conspecifics |  | Damaging behaviour to conspecifics |  | Damaging behaviour to conspecifics |  |
| 4 | Grouping of pigs of different age and/or size | Soft tissue lesions and integument damage | Inadequate pest control |  | Grouping of pigs of different age and/or size |  | Grouping of pigs of different age and/or size |  | Grouping of pigs of different age and/or size |  | Grouping of pigs of different age and/or size |  |
| 5 | High stocking density |  | Inappropriate diet |  | High stocking density |  | High stocking density |  | High stocking density |  | High stocking density |  |
| 6 | Inadequate length or width of pens |  | Inappropriate environmental enrichment |  | Inadequate access to udder due to bars etc. |  | Inappropriate environmental enrichment |  | Inappropriate environmental enrichment |  | Inappropriate environmental enrichment |  |
| 7 | Inadequate slot dimensions in relation to foot dimensions |  | Inappropriate materials in feed |  | Inappropriate environmental enrichment |  | Insufficient environmental enrichment |  | Insufficient environmental enrichment |  | Insufficient environmental enrichment |  |
| 8 | Inappropriate environmental enrichment |  | Inappropriate perforation of slats to keep pen clean from manure and urine |  | Insufficient environmental enrichment |  | Insufficient foraging/exploration/enrichment material |  | Lack of elasticity of hard flooring material |  | Insufficient foraging/exploration/enrichment material |  |
| 9 | Insufficient environmental enrichment |  | Inappropriate water quality |  | Insufficient foraging/exploration/enrichment material |  | Lack of overall space |  | Lack of overall space |  | Lack of elasticity of hard flooring material |  |
| 10 | Insufficient foraging/exploration/enrichment material |  | Insufficient environmental enrichment |  | Lack of overall space |  | Large group size |  | Large group size |  | Lack of overall space |  |
| 11 | Insufficient solid floor |  | Insufficient foraging/exploration/enrichment material |  | Large group size |  | Poor flooring conditions |  | Poor quality pen design (lack of barriers/dividers in pens) |  | Large group size |  |
| 12 | Insufficient space allowance |  | Lack of overall space |  | Poor quality pen design (lack of barriers/dividers in pens) |  | Poor quality pen design (lack of barriers/dividers in pens) |  | Poor quality pen design (open sides between adjacent pens) |  | Poor flooring conditions |  |
| 13 | Lack of elasticity of hard flooring material |  | Poor quality pen design (lack of barriers/dividers in pens) |  | Poor quality pen design (open sides between adjacent pens) |  | Poor quality pen design (open sides between adjacent pens) |  |  |  |  |  |
| 14 | Lack of overall space |  | Poor quality pen design (open sides between adjacent pens) |  |  |  |  |  |  |  |  |  |
| 15 | Large group size |  |  |  |  |  |  |  |  |  |  |  |
| 16 | No bedding |  |  |  |  |  |  |  |  |  |  |  |
| 17 | Poor flooring conditions |  |  |  |  |  |  |  |  |  |  |  |
| 18 | Poor housing design and allocation of resources |  |  |  |  |  |  |  |  |  |  |  |
