# Supplementary material for: Optimising the selection of welfare indicators in farm animals
Source: Front Vet Sci. 2025 Oct 28;12:1661470. doi: 10.3389/fvets.2025.1661470 (PMC12604357; doi:10.3389/fvets.2025.1661470)
Supplement: Supplementary file 6 [file Supplementary_file_6.docx]

Supplementary Material 6

| 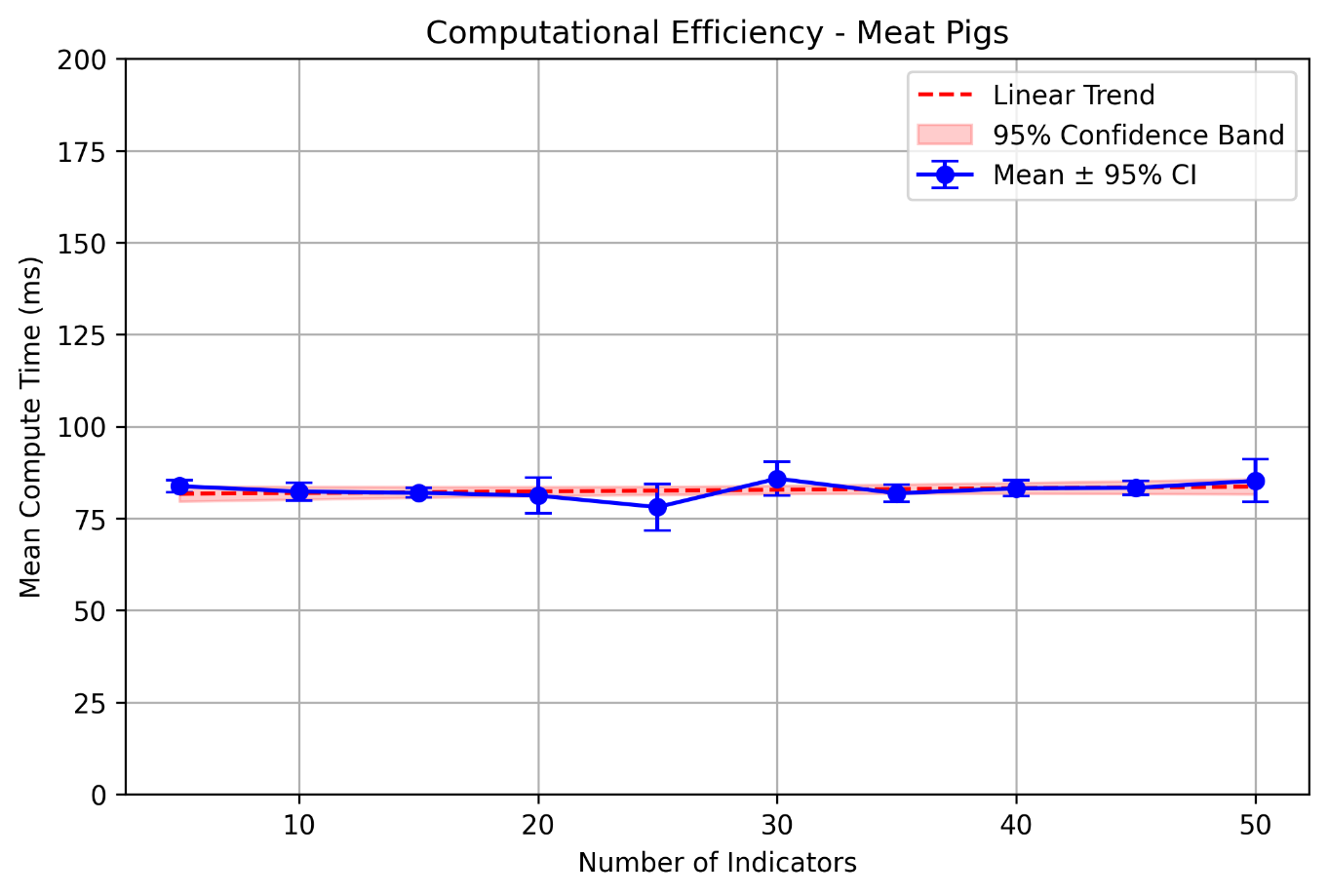 |
| --- |
| **Supplementary material 6 (A):** *The mean computation time (in milliseconds) required to run the enhanced algorithm using SCIP for meat pigs, plotted against the number of welfare indicators considered. The blue line with error bars represents the mean computation time with 95% confidence intervals across repeated runs. A red dashed line indicates the linear trend, with a shaded band representing its 95% confidence interval.* |

| 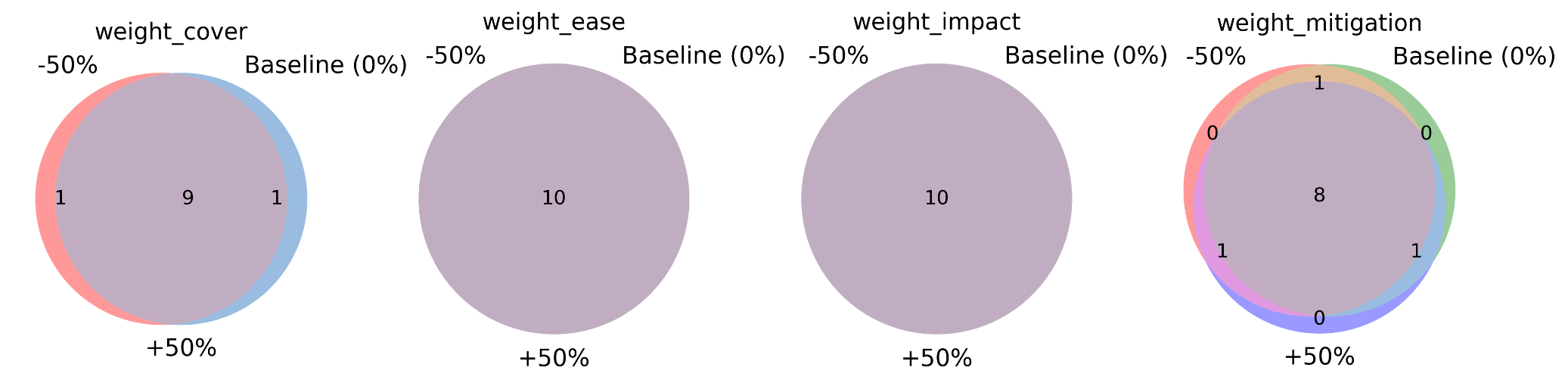 |
| --- |
| **Supplementary Material 6 (B):** *The effect of introducing set perturbations (-50% (red circles), 0 (green circles) & +50% (purple circles)) into the weighting factor for: a) Coverage (ω_coverage), b) Ease of indicator use (ω_easiness), c) Impact of welfare consequence (ω_impact), and d) Ease of hazard mitigation (ω_mitigation) for meat pigs. All weighting factors were initially set at 1.0, with the exception of the one that was to be manipulated. The maximum number of indicators was constrained to 10. Plotted values represent the number of shared indicators in each perturbation condition.* |
